# Adsorption-Induced Optical Modulation in ZIF Thin Film Stacks with Distinct Order for Photonic Crystal Applications

**DOI:** 10.1021/acsami.5c02529

**Published:** 2025-05-01

**Authors:** Nils Christian Keppler, Lukas Steinbach, Johanna Fricke, Adrian Hannebauer, Erik Rohloff, Andreas Schaate, Peter Behrens

**Affiliations:** a Institute of Inorganic Chemistry, Leibniz University Hannover, Hannover 30167, Germany; b Cluster of Excellence PhoenixD (Photonics, Optics and Engineering − Innovation Across Disciplines), Leibniz University Hannover, Hannover 30167, Germany

**Keywords:** metal−organic frameworks (MOFs), refractive index, optical materials, MOF thin films, zeolitic imidazolate frameworks (ZIFs)

## Abstract

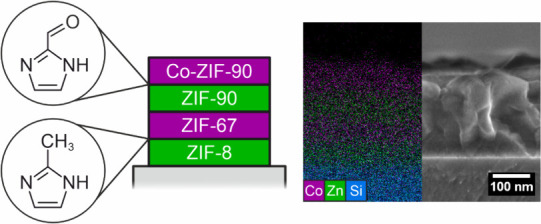

Due to their high chemical and thermal stability, zeolitic imidazolate frameworks (ZIFs) are interesting materials for various applications. To adapt ZIFs for new applications, such as optics, it is necessary to achieve precise shaping and control over film formation. Thin films are ideal for this purpose, as several of them are commonly used in optical devices. Here, we present a variety of different ZIF thin films that are accessible by applying a ZIF-8 seeding layer approach. High-quality ZIF-8 thin films are used as a seeding layer for the rapid production of other ZIF layers. All ZIFs are provided in high optical quality and have been characterized with respect to their optical properties. The presented thin films can also be fabricated as stacked layers with the desired order of different ZIFs to form photonic crystal structures, such as Bragg-stacks. Depending on the system, the fabrication of the stacked layers takes only a few hours. Finally, the thin films are loaded with guest molecules to influence their optical properties. Subsequent selective loading of the pores in ZIF 8/ZIF 90 stacks was achieved by using guest molecules of different polarity.

## 1 Introduction

Metal–organic frameworks (MOFs) are hybrid materials formed by connecting inorganic building units, such as metal ions or metal-oxo clusters, with organic linker molecules to create three-dimensional networks.^1,2^ Their ultrahigh porosity^3^ and tunability^4−7^ enable applications in sensing,^8,9^ catalysis,^10,11^ gas storage^12−14^ and gas separation.^15−17^ More recently, MOFs have gained interest in biomedical technologies,^18−20^ electronics, and optics.^21−24^ In optics, their tunable refractive index^25−29^ make them valuable for optical sensing and adaptive coatings.^30^

Many MOFs suffer from low chemical and thermal stability.^31^ To address this, stable subclasses such as zirconium-based MOFs and zeolitic imidazolate frameworks (ZIFs) have been developed.^32,33^ ZIFs share structural features with zeolites and are often considered their MOF analogues. The same structural elements and structure types occur as in zeolitic materials. Here, we deal exclusively with ZIFs of the sodalite (**sod)** structure type, one of the simplest zeolitic structures. The unit cell of ZIF-8, the most basic ZIF representative, is cubic (space group *I*4̅3*m*) and consists of sodalite cages formed by a three-dimensional network of tetrahedrally coordinated Zn atoms, connected by imidazolates, typically enclosing pores with a diameter of about 11.6 Å for the ZIF-8.^32^ These materials allow fine-tuning of chemical properties via linker functionalization and the use of different metal centers.^34−37^ Additionally, heterostructures, like core–shell particles of ZIFs with different metal ions or linkers, are well established.^38,39^

Substrate-supported MOF thin films are essential for integrating MOFs into optical devices.^34^ Various coating techniques, including spin, dip or spray coating of nanoparticles,^40−42^ microfluidic growth,^42^ molecular layer deposition (MLD),^43,44^ liquid-phase epitaxy (LPE), layer-by-layer growth (LbL)^36^ or solvothermal growth^45^ have been developed. Their major drawbacks are poor film qualities, long preparation times, or even both.^23,35,46,47^

Thin films for optical applications, such as Bragg-stacks and other photonic crystals, require specific sequences of materials with differing optical characteristics, such as refractive index or transmittivity.^41,48,49^ Bragg-stacks, for instance, consist of alternating layers of materials with different refractive indices, ensuring the reflection of specific wavelengths of light.^49^ The photonic stop band,^50^ the wavelength range in which the mirror reflects, depends on layer thickness, number of layers and refractive index difference.^49^ Bragg reflectors are used in optical devices like mirrors and filters^51^ and have gained interest for light confinement in emitting and absorbing devices, including solar cells and LEDs.^52,53^ While traditionally made of dense nitride^54^ or oxide^55^ materials, meso- and microporous materials^41,51^ are gaining attention for sensory applications. Porous, responsive Bragg stacks typically rely on non-MOF materials such as TiO_2_ to obtain film stacks of optical quality.^41,42,45,56^ For this reason, we focus on preparing MOF-on-MOF layers with optical quality, as MOFs offer greater tunability of optical properties compared to the previously mentioned materials.

Layered MOF systems have been studied extensively, but their fabrication remains time-consuming, typically taking about a week.^40,46,47,57−59^ To overcome this, we focus on synthesizing zeolitic imidazolate frameworks using a cycle-based direct growth method, initially introduced by the Hupp group for ZIF-8, to accelerate MOF thin film and stack synthesis.^25,30,34^ This method is compatible with various solvent-resistant substrates, including silicon wafers, glass and PMMA. For substrates unsuitable for direct growth, surface functionalization with self-assembling monolayers (SAMs) offers an alternative.^43^ As demonstrated in our previous work, ZIF-8 thin films can serve as seeding layers for the fabrication of layer stacks consisting entirely of different ZIF thin films.^30^ A first example of this approach was shown for Co-ZIF-90, which we found to be optically sensitive to water vapor.^30^ Furthermore, in order to combine this sensitivity of the ZIF films with the optical activity of other materials, the lattercan be incorporated into the films, as was shown for 2D semiconducting nanoplatelets.^60^

In this paper, we demonstrate that the seeding layer approach can be applied to various ZIFs crystallizing in **sod** topology, enabling the fabrication of optically transparent film stacks with a defined order of layers. Through precise refractive index determination via ellipsometry and the targeted growth of different ZIF derivatives, we establish a systematic approach to control optical and adsorptive properties, laying the foundation for a database of ZIF derivatives with distinct characteristics.

By utilizing the porosity of ZIF thin films, optical characteristics, such as transmittance can be modulated through sequential guest loading. These switchable layer sequences can be used to design optical components, including thin films and Bragg-mirrors, grown under mild, solvent-based conditions in a short time, presenting an alternative to conventional thin film synthesis.

## 2 Methods

### 2.1 Materials

Zinc(II) nitrate hexahydrate (98%, Sigma-Aldrich, Zn(NO_3_)_2_ · 6 H_2_O), zinc(II) acetate dihydrate (98%, abcr, Zn(OAc)_2_ · 2 H_2_O), cobalt(II) nitrate hexahydrate (98%, Roth, Co(NO_3_)_2_ · 6 H_2_O), cobalt(II) acetate tetrahydrate (98%, Sigma-Aldrich, Co(OAc)_2_ · 4 H_2_O), 2-methylimidazole (99%, Sigma-Aldrich, HmIm), imidazole-2-carboxaldehyde (97%, TCI, HImCA), 2-(trifluoromethyl)-imidazole (99.9%, BLDpharm, HImCF_3_), methanol (99.5%, Roth), ethanol (99.8%, dry, Fisher-Scientific), dimethylformamide (99.8%, Sigma-Aldrich, DMF), sulfuric acid (96%, Roth) and hydrogen peroxide (35%, Roth) were used without further purification.

Sb-doped silicon wafers with (111)-orientation from Microchemicals were used as substrates for all syntheses referred as “on silicon”. The wafers were prepared for most of the applied characterizations methods by cutting pieces of 1 × 1 cm. For the thin film growth “on glass”, microscopy glass slides from VWR were cut into pieces of 21.5 × 26 mm (for UV–vis measurements) or 15 × 15 mm (for photographs). All substrates were cleaned in a freshly prepared mixture of sulfuric acid and hydrogen peroxide (2:1) for at least 10 min. Subsequently, they were washed with deionized water and methanol. Cleaned substrates were used immediately.

### 2.2 Synthesis of ZIF Thin Films with Cycle-Based Growth

All reactions were carried out at room temperature. Stock solutions were prepared at the concentrations described in Table S1 (ESI). All solutions were produced by dissolving the precursor in the mentioned solvent at room temperature while stirring, except for the solutions of imidazole-2-carbaldehyde, which had to be dissolved at 70 °C for ca. 30 to 60 min until a clear solution was achieved, depending on the concentration.

ZIF-67, Zn(ImCF_3_)_2_, ZIF-90 and Co-ZIF-90 thin films were prepared on ZIF-8 thin films as seeding layer. The thin film growth consists of the following two steps: (1) A ZIF-8 seeding layer was prepared according to Lu and co-workers^34^ by incubating the previously cleaned substrate in a freshly prepared 1:1 mixture of the precursor solutions for 30 min. (2) The substrate from step (1) was removed from the ZIF-8 synthesis solution, rinsed with methanol and directly reincubated in a 1:1 mixture of the precursor solutions for ZIF-8 or another ZIF listed in Table S1. It is important to immediately reincubate the substrate after rinsing, as cracks may from due to the intermediate drying between steps (1) and (2), as previously described in the advanced synthesis of ZIF-8 films.^25^ The substrate was removed from the synthesis solution after 30 min and step (2) was repeated with the same or another ZIF until the desired thin film system was manufactured. The obtained coated substrates were air-dried after the final synthesis cycle.

### 2.3 Synthesis of ZIF-8 and ZIF-90 Powder Samples

ZIF-8 powder samples were synthesized by mixing solutions of the precursors zinc nitrate-hexahydrate (25 mM) and 2-methylimidazole (200 mM) in methanol.^61^ The mixture, which became cloudy after a few minutes, was stirred at room temperature for 2 h. Subsequently, the crystals were collected by centrifugation at 5000 rpm for 10 min. They were washed with ethanol twice and dried under reduced pressure at room temperature. ZIF-8 was obtained as a white crystalline powder.

ZIF-90 powder samples were synthesized analogously using different precursor solutions: Zinc acetate-dihydrate (25 mM) in methanol and 100 mM HImCA (100 mM) in DMF. The synthesis solution became cloudy immediately after mixing the two solutions at room temperature. After 2 h of stirring at room temperature, the particles were collected by centrifugation at 5000 rpm for 10 min. The particles were washed with methanol and ethanol and dried under reduced pressure at room temperature. ZIF-90 was obtained as a light-yellow crystalline powder.

### 2.4 Characterization of Thin Films and Powders

X-ray diffraction (XRD) measurements on ZIF-8 thin films were carried out with an X-ray diffractometer from STOE working in Bragg–Brentano geometry. An Iso-Debyeflex 3003 from Malvern Panalytical was used for the generation of X-rays at 40 kV and 30 mA, delivering Cu*K*α_1_ radiation. Measurements were carried out between 5 and 30° 2θ with a step size of 0.02° 2θ and a measurement time of eight seconds per step.

A SE800 spectroscopic ellipsometer from Sentech with a spectral range from 400 to 850 nm was used for the ellipsometry measurements. All measurements were performed at an angle of incidence of 70°. Ellipsometry data were fitted with a Cauchy dispersion model (ZIF-8 and Zn(ImCF_3_)_2_) or a Tauc-Lorentz model (ZIF-90, Co-ZIF-90 and ZIF-67). More information on the models used to evaluate the ellipsometry data is provided in the ESI (see Table S2 and the explanatory text). To investigate the refractive index of ZIF-8 and ZIF-90- in a controlled gas atmosphere, ellipsometry measurements were performed in an atmosphere containing methanol using mass-flow controllers (EL-FLOW select, Bronkhorst). A detailed description of the experiment is given in the ESI (Figure S14 and explanatory text).

Scanning electron microscopy (SEM) images of coated silicon substrates were prepared by attaching samples on SEM aluminum substrate holders with conductive carbon tape (for top-view images). Electrical contact of the thin films was ensured by the use of a conductive silver lacquer. Cross-section images were obtained by cutting a predefined breaking point on the backside of the substrate with a diamond engraving pen and breaking them afterward. The substrates were fixed in an aluminum holder with the breaking edge up. All images were captured using a Regulus SU8200 (Hitachi). Images were typically recorded at a voltage of 2 kV and a current of 10 μA.

UV–vis spectra on coated glass substrates were recorded in transmission with a Cary 4000 (Agilent Technologies) in the spectral range from 300 to 800 nm using the software “Scan”. We used a home-built UV–vis measurement cell with quartz glass windows mounted tightly in the spectrometer providing gas inlet and outlet. The atmosphere was controlled by changing the gas flow through the measurement cell. The gas flows were controlled by two mass-flow controllers (EL-FLOW select, Bronkhorst). A detailed description of the experiment is given in the ESI (Figure S14 and explanatory text). Reflectance spectra were measured using a Cary 4000, equipped with a Praying Mantis Diffuse Reflectance Accessory (from Agilent Technologies). The samples were grown and measured on silicon wafers, with a bare silicon wafer serving as background.

The X-ray photoelectron spectroscopy (XPS) depth profiling was performed using a PHI VersaProbe III (Physical Electronics GmbH) XPS system. The chemical state of the material was analyzed using Al K_α_ X-rays (1486.7 eV, 50 W, 15 kV) with an X-ray spot size of 200 μm in diameter; dual-beam charge neutralization (a 1 V electron beam and a 7 V Ar^+^-ion beam) was applied during the measurements. For the sputtering phase (6 s for each sputtering step), a 20 kV argon gas cluster ion beam with a sputter grid size of 2 mm × 2 mm was used. The acquired data were processed and evaluated using the MultiPak Software (ULVAC-PHI).

Static sorption isotherms for the adsorption of water, methanol and ethanol in ZIF-8 and ZIF-90 powder samples were recorded on a Vapor 100C (3P instruments). The vapor source was purified three times using a freeze-drying method. The temperature of the vapor source was then maintained constant at 40 °C for water and 30 °C for methanol and ethanol. The powder samples (40 mg) were activated at 120 °C for 20 h under vacuum. Additional *in situ* activation at 120 °C for 4 h was performed prior to measurement. Isotherms were recorded at 25 °C.

## 3 Results and Discussion

**Figure 1:**
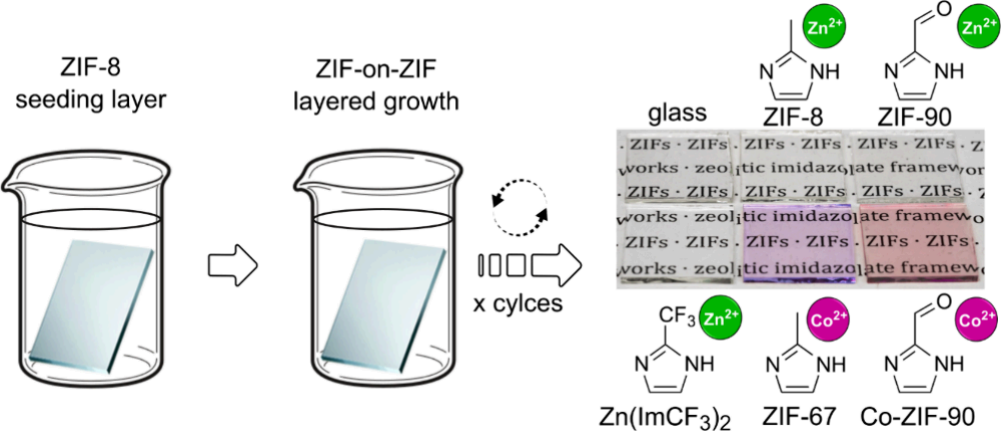
Schematic overview of the layer based direct growth method. First, a ZIF-8 seeding layer is grown. Subsequently, various layers of the five derivatives shown on the right side can be grown on top until the desired thickness or layer sequence is achieved. The ZIF films on the right side are grown on glass substrates and each consists of one seeding layer of ZIF-8, followed by two consecutive layers of the respective derivative.

The synthesis of ZIF thin films was performed on the basis of the cycle-based synthesis approach first described by the Hupp group^34^ and developed further by us (see Experimental section for details), as schematically depicted in Figure 1.^25,30^ The first seeding layer always needs to be ZIF-8. In order to obtain thicker films or ZIF-on-ZIF coatings, additional synthesis cycles for the same or a different ZIF can be performed until the desired film or film stack is obtained.

We developed and optimized synthesis protocols for ZIF-67, ZIF-90 and Zn(ImCF_3_)_2_ which will not be discussed in detail here, see ESI Table S1 and explanatory text for more details. For ZIF-8 and Co-ZIF-90, previously reported optimized conditions were used.^30^ Further information on the synthesis conditions can be found in the Methods section and in the ESI (Table S1). It should be noted that Zn(ImCF_3_)_2_ has not been reported before, only ZIF-318 has been reported as a zinc-based ZIF with two different linkers, HmIm and HImCF_3_ in a ratio of 1:1. Without the addition of HmIm, no ZIF could be synthesized using HImCF_3_ alone.^62^ Applying the ZIF-8 seeding layer approach previously described by our group for Co-ZIF-90,^30^ we succeeded in growing a ZIF layer with **sod** topology with HImCF_3_ as the sole linker.

Photographs of the resulting thin films on glass substrates are shown in Figure 1. All samples feature one ZIF-8 seeding layer followed by two consecutive layers of a second ZIF. All films are transparent and appear clear to the naked eye, without visible scattering, indicating high optical quality. As expected for tetrahedrally coordinated cobalt(II)-ions, the cobalt-based ZIFs (ZIF-67 and Co-ZIF-90) are colored due to d-d-transitions.^63,64^ In contrast, the zinc-based ZIFs (ZIF-8, ZIF-90 and Zn(ImCF_3_)_2_) are colorless.

### 3.1 Characterization of Substrate-Supported ZIF Thin Films

**Figure 2:**
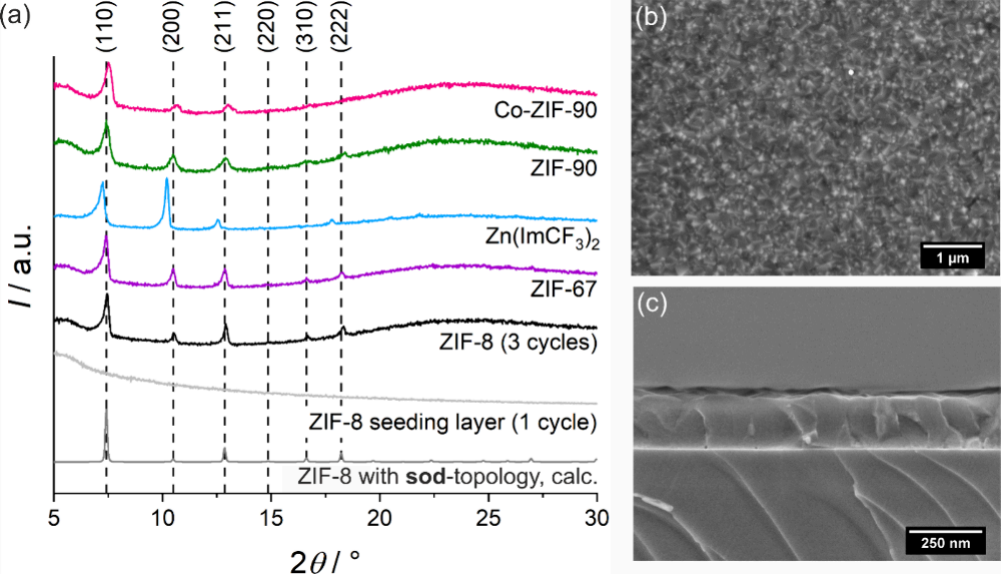
(a) XRD patterns of ZIF thin films on glass substrates. Thin films of ZIF-67, Zn(ImCF_3_)_2_, ZIF-90 and Co-ZIF-90 (each with two deposition cycles) were grown on ZIF-8 seeding layers (one deposition cycle of ZIF-8). A calculated XRD pattern of ZIF-8 is shown as a reference, with peak position and corresponding lattice planes labeled.^32^ (b) SEM top view and (c) cross-section images of a ZIF-8 film (three cycles) on a silicon substrate.

XRD patterns of all ZIF thin films that are used in this study are shown in Figure 2a. As for the photographs in Figure 1b, all thin films were synthesized on glass substrates, as indicated by the signal of amorphous silica in the background of the diffraction patterns. The thin film systems measured are all including one seeding layer of ZIF-8 and two consecutive layers of the ZIF derivative, as described. The presence of reflections in all diffractograms (with the exception of the ZIF-8 seeding layer, which is too thin to show reflections), indicates that the thin films are crystalline. The positions of the reflections in all XRD patterns demonstrate a high degree of agreement with the calculated pattern of a ZIF with **sod** topology (ZIF-8 was used as reference). Small deviations in the positions of the reflections can be attributed to slight variations in the cell parameters of the ZIF derivatives.^32,62,65^ The increased peak width suggests the presence of crystallites in the nanometer range, indicating that the films are polycrystalline. The broadening of the peaks may also be attributed, at least in part, to lattice strain, considering that the cell parameters of the other four derivatives differ from those of ZIF-8. The altered intensity distribution further indicates a preferred orientation of the thin films. In particular, the (200) reflection exhibits increased intensity, which suggests a preferred orientation of crystallites along this direction.

Figure 2 also shows representative SEM images of ZIF-8 in top view (b) and cross-section (c). SEM images of the other films are included in the ESI Figure S1. The top view image reveals that the film is polycrystalline, as was already expected from the XRD pattern. The crystallites are nanometer-sized, homogeneous and densely intergrown. This dense intergrowth is further confirmed by the cross-section image, where no individual crystallites are visible. The film appears as a dense layer with a thickness of approximately 300 nm. Furthermore, no cracks are observed between individual growth cycles.

The SEM images of the other derivatives (ESI Figure S1) show similar results. Only the thin films of ZIF-90 exhibit narrow cracks, possibly due to subtle lattice mismatch between ZIF-8 and ZIF-90, which could induce strain during growth. All other ZIF films are crack-free and show only minor inhomogeneities on the surface - mostly small crystals with diameters well below 1 μm. The cross-section images confirm that all films are fully intergrown, with no visible transition between the ZIF-8 seeding layer and the overgrown derivative layer. The surfaces of all films are sufficiently flat for the intended investigations and all ZIF thin films grew to a similar thickness of about 250 nm (including seeding layer of approximately 50–100 nm).^30^ An exception is the Zn(ImCF_3_)_2_ layer, which is much thicker (about 500 nm including the seeding layer) due to its higher growth rate compared to the other ZIFs.

To avoid crack-formation in ZIF-90, the number of deposition cycles can be reduced. Performing only one deposition cycle instead of two leads to lower lattice stress and results in a crack free thin film, as shown in ESI Figure S2.

**Figure 3:**
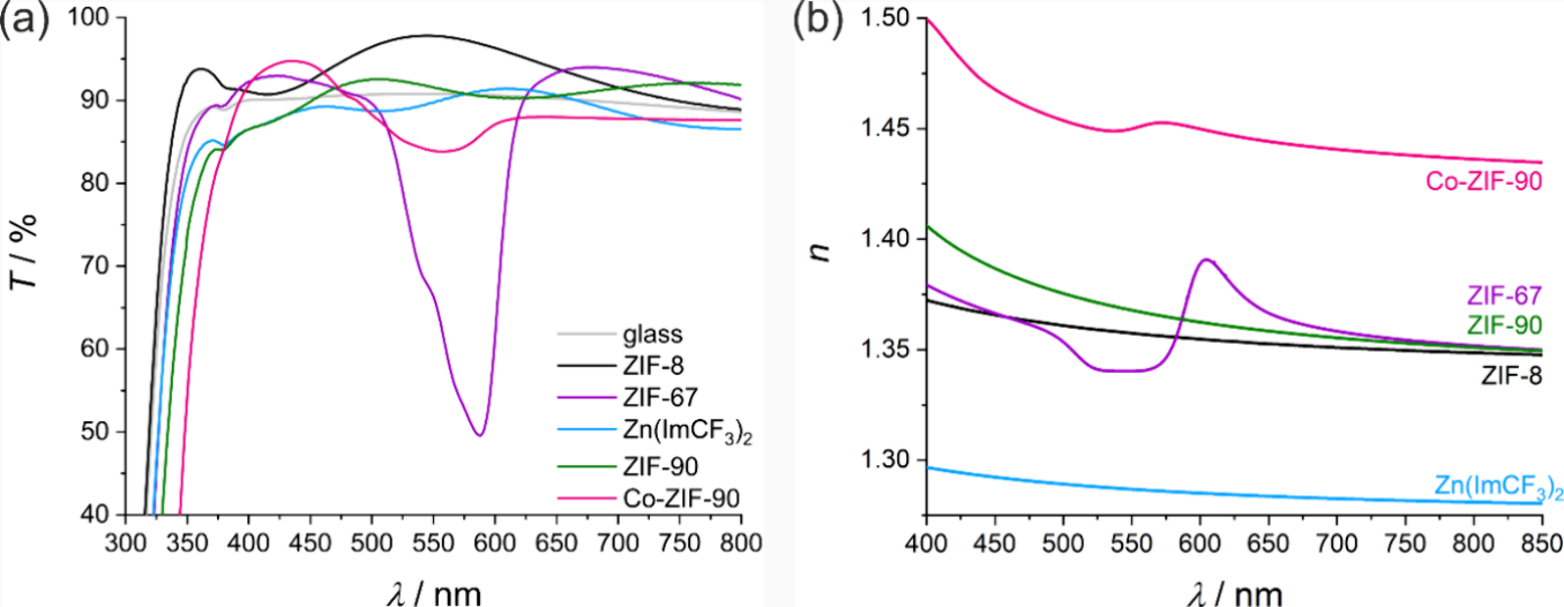
(a) UV–vis transmission spectra of ZIF thin films measured on glass substrates and (b) refractive index dispersions of ZIF thin films measured on silicon wafers. The thin films of ZIF-67, Zn(ImCF_3_)_2_, ZIF-90 and Co-ZIF-90 (two deposition cycles for the each ZIF) were prepared on ZIF-8 seeding layers (one deposition cycle of ZIF-8). The UV–vis transmission spectrum of uncoated glass is shown in (a) as reference. All UV–vis measurements were performed in dry argon atmosphere to avoid loading of the pores with guests from the surrounding atmosphere (especially moisture). A dry argon atmosphere was also used for the background measurement.

UV–vis transmission spectra of all thin films are shown in Figure 3a. The transmission spectrum of uncoated glass is shown as a reference, and it can be seen, that the glass used in our experiments completely absorbs light with a wavelength below 350 nm. This absorption is also seen in the transmission spectra of the ZIF coated glass slides. Absorption bands between 500 and 600 nm are present in the transmission spectra of ZIF-67 and Co-ZIF-90, which are caused by the cobalt(II) ions in these frameworks.^63,64^ The transmission spectra of the other thin films show waves that are typical for colorless optical thin films and that can be attributed to interference phenomena.^34^ The positions and widths of the waves vary with the thickness and refractive index of the thin films.^25^ In all spectra, there are areas where the transmission through the ZIF-coated glass exceeds that of the uncoated glass slide – across the entire spectrum for ZIF-8. In this case, antireflectivity (AR) is achieved due to the low scattering, high transmission, and the low refractive indices of the ZIF thin films compared to glass. This confirms the high optical quality of the films. The antireflective behavior can be seen on the UV–vis reflection spectra, as illustrated in Figure S3 in the ESI. The spectra of these thin films were measured on the (111) surface of a silicon wafer, with a blank wafer serving as baseline. In this case, the AR behavior is even more pronounced in comparison to the spectra on glass, which can be attributed to the higher refractive index of the silicone compared to glass. For ZIF-67 and Co-ZIF-90, absorption features between 500 and 600 nm are visible again.

The refractive index dispersions of all investigated ZIF thin films were measured by ellipsometry and are shown in Figure 3b. Low refractive indices below 1.5 were measured for all ZIFs. These small values are caused by the porosity of the MOF films.^25,34^ If the refractive index *n*_AR_ of a thin film follows the condition *n*_air_ < *n*_AR_ < *n*_substrate_, antireflectivity can be observed in some cases.^66^ ZIF-8, ZIF-90 and Zn(ImCF_3_)_2_ show normal refractive index dispersions that are typical for materials that are not absorbing in the visible light spectrum and show the characteristic decrease in refractive index with increasing wavelength. The higher refractive index of ZIF-90 at lower wavelength compared to that of ZIF-8 can be explained by the smaller band gap of ZIF-90 resulting in a higher refractive index near the band gap.^67,68^ Zn(ImCF_3_)_2_ has a lower refractive index compared to ZIF-8 which is typical for fluorinated molecules because of the lower polarizability that is caused by the high electron affinity of fluorine (typical examples are benzene and hexafluoro benzene with refractive indices of 1.50 and 1.38 at 589 nm).^69^ The refractive index dispersions of ZIF-67 and Co-ZIF-90 show absorption bands between 500 and 650 nm that are caused by the cobalt(II) ions. ZIF-67 shows a much higher absorption, as expected from the UV–vis transmission spectra in Figure 3a.

### 3.2 Synthesis and Characterization of Mixed ZIF Thin Film Stacks

The results presented so far show that we are able to fabricate ZIF thin films of high optical quality that can be characterized well by various methods such as ellipsometry, UV–vis spectroscopy, XRD, and SEM. All thin films were grown on top of a ZIF-8 seeding layer showing that the different ZIFs with **sod** topology are compatible with ZIF-8 due to similar cell parameters, which becomes evident from the XRDs shown in Figure 2a. In this chapter it is shown that mixed ZIF stacks of more than two different layers can also be realized.

Two different mixed ZIF stacks were fabricated as model systems: one having three ZIF layers (ZIF-8/Co-ZIF-90/ZIF-90), shown in the manuscript, the other having four ZIF layers (ZIF-8/ZIF-67/ZIF-90/Co-ZIF-90) shown in the ESI. The mixed ZIF stacks were characterized by SEM, EDX mapping, XPS, and UV–vis. Ellipsometry was used to determine the refractive indices of the individual layers in the stacked system in order to compare the obtained values to these of the simple systems consisting of a single ZIF on a seeding layer. Additionally, photographs of the resulting films on glass substrates were taken to show the color and transparency by the naked eye.

**Figure 4:**
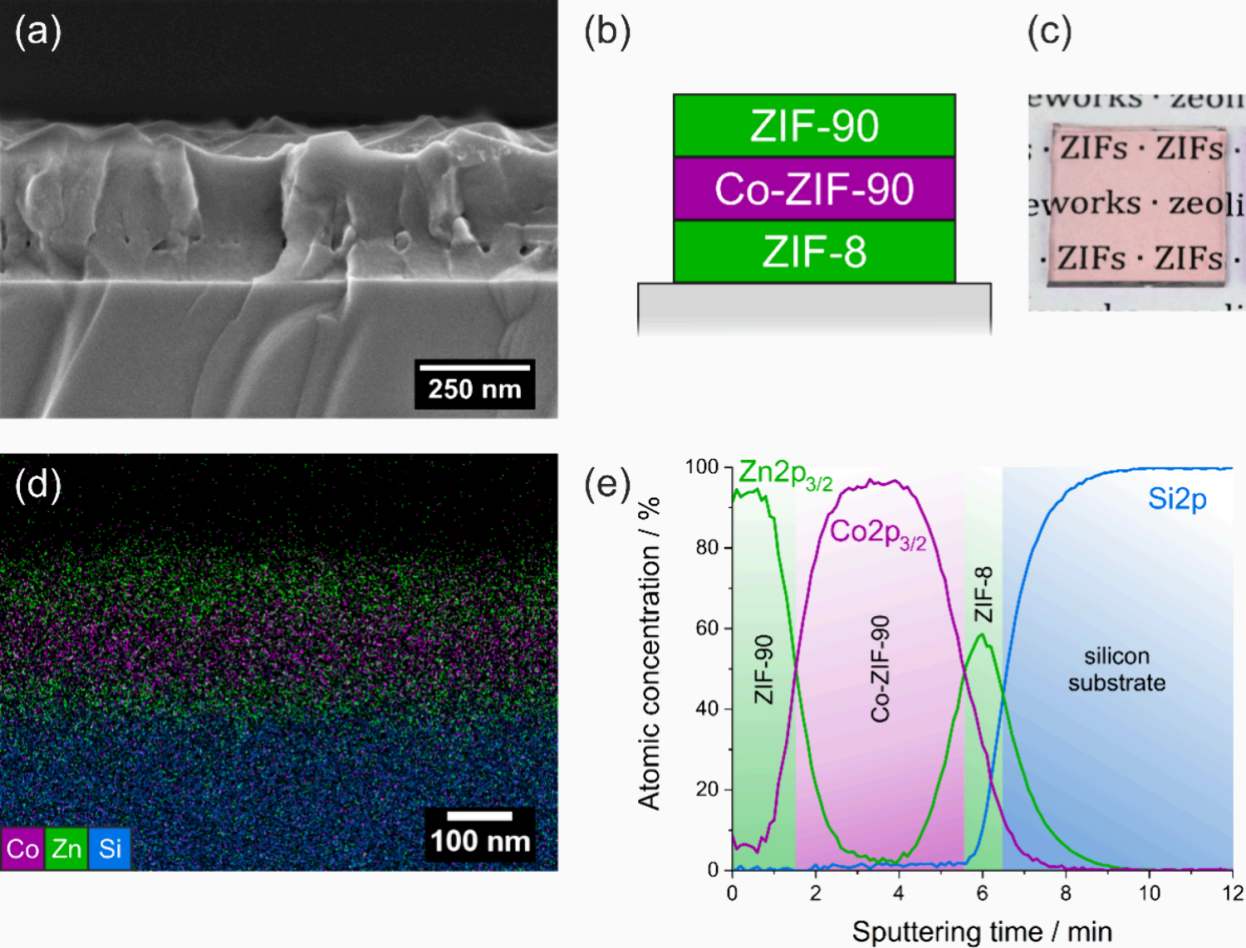
(a) Cross-section SEM image of the three layer stack consisting of ZIF-8/Co-ZIF-90/ZIF-90, fabricated on a silicon substrate. (b) Schematic representation of the layer sequence. (c) photography of the thin film stack coated on glass. (d) EDX mapping of a cross-section and (e) a XPS depth profile measurement on samples fabricated on silicon substrates.

Characterizations for the three-layered system via SEM and ellipsometry measurements are shown in Figure 4a, for the four-layered system the corresponding measurements are found in the ESI (Figure S4a). The cross-sectional SEM image of the (ZIF-8/Co-ZIF-90/ZIF-90) film shows a uniform intergrowth with no visible transitions at the interfaces and a total thickness of around 300 nm. A schematic illustration of the film stack is depicted in Figure 4b, with the equivalent depiction for the four-layered system in Figure S4b. A photograph of the stack is shown in Figure 4c, for the four layered system in Figure S4c, respectively. The mixed ZIF stack appears highly transparent to the naked eye, as evidenced by the good readability of the printed text beneath the substrates. Reflection behavior, measured via UV–vis reflectance spectra for the three- and four-layered system are shown in Figure S5 in the ESI. Antireflection effects can also be seen here, as with the simpler samples before.

To investigate the assumed layer sequence in the mixed stacks, EDX mapping and XPS depth profiling were performed (shown in Figure 4d and 4e). In our model systems, zinc- and cobalt-containing ZIFs were deposited in an alternating fashion. This allows elemental analysis for zinc and cobalt to determine the locations of the zinc containing ZIF-8 and ZIF-90 as well as the cobalt containing ZIF-67 and Co-ZIF-90.

Cross-sectional EDX mappings show signals for silicon, zinc, and cobalt. The Silicon signal is predominantly detected at the bottom of the image, originating from the silicon substrate, while the cobalt and zinc signals are caused by the ZIF thin films. The EDX image of the three-layered stack shows zinc signals at the bottom and top corresponding to the ZIF-8 (bottom) and ZIF-90 (top) layers. In the EDX image of the four–layered stack, four distinct layers are observed – two containing zinc and tow containing cobalt. Zinc is found at the bottom of the stack (ZIF-8) and in the third layer from the bottom (ZIF-90), while cobalt is found in the second (ZIF-67) and fourth (Co-ZIF-90) layers from the bottom. These results show that distinct alternating layers have been synthesized in both cases. The measurement for the four-layered sample is depicted in Figure S4d in the ESI.

XPS depth profiling of the atomic concentrations of zinc, cobalt and silicon, which are shown in Figure 4e for the three-layered system and Figure S4e for the four-layered system, are leading to results consistent with those obtained from the EDX measurements.

During XPS depth profiling, XPS measurements and argon cluster sputtering alternate to progressively remove surface material, with elemental analysis performed between cycles. Increasing sputtering time reveals the elemental composition of deeper layers in the ZIF stack down to the substrate.

In the three-layered sample shown in Figure 4e, zinc (from ZIF-90) is initially the dominant transition metal. As sputtering proceeds, cobalt (from Co-ZIF-90) increases and eventually overtakes zinc, marking the interface where their signal curves intersect. With further sputtering, the cobalt signal declines to zero, while zinc rises again due to the underlying ZIF-8 layer. Finally, once the entire ZIF stack is removed, the silicon substrate becomes dominant and ultimately the only detectable element. These results demonstrate that the thin film stack of ZIF-8, Co-ZIF-90 and ZIF-90 was successfully synthesized in the intended order.

A similar depth profile is observed for the four-layer stack, featuring two cobalt-containing and two zinc-containing layers, as shown in Figure S4e. The XPS depth profiling are in agreement with the EDX data presented earlier and further confirm the successful synthesis of three- and four-layered systems. The corresponding XPS survey and sputtering time-dependent spectra are shown for the three-layer system in Figures S7 and S8, for the four-layered system in Figures S9 and S10 in the ESI.

**Figure 5:**
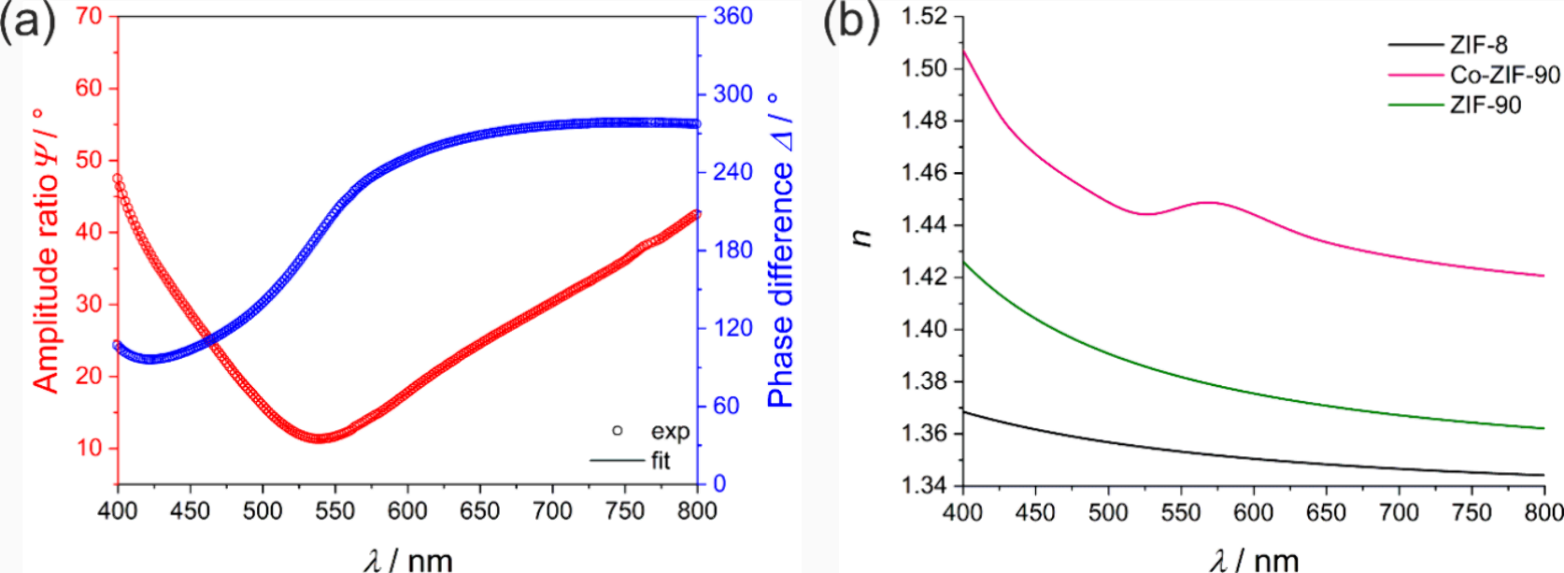
(a) Ellipsometry fit of the ZIF-8/Co-ZIF-90/ZIF-90 stack on silicon. The experimental values (circles) and fit results (line) for the ellipsometry parameters Ψ and Δ are shown. (b) Shows the resulting refractive index dispersions derived from the ellipsometry fits.

Results of the ellipsometry measurements are presented in Figure 5a and b and for the four layered system it can be found in ESI Figure S5a and b. Ellipsometry measurements were performed to validate the correct ordering of the thin films and to verify that the refractive indices are comparable to those shown in Figure 3b. A good agreement between the experimental data (circles) and fitted data (line) of Ψ and Δ can be seen for the ellipsometry measurement of the three-layered stack (Figure 5a). The refractive indices for all thin films resulting from these fits are shown in Figure 5b. They are in good agreement with the refractive indices measured on simpler systems with only two different layers (see Figure 3b), further demonstrating the high optical quality of the films, as even multilayer systems exhibit no signs of scattering or other interfering optical effects. Furthermore, this shows that little to no postsynthetic exchange of linkers or cations takes place between the individual layers during the cyclic growth of the films, as this would have a direct influence on the refractive index of the films shown. This again confirms that the successive growth cycles are deposited as separate layers.

### 3.3 Optical Modulation of ZIF Thin Film Stacks Using the Model System ZIF-8/ZIF-90

We have shown, that subsequent growth of different **sod** type ZIFs is possible via direct growth in multiple cycles. The differing functional groups of the imidazole linkers used in this study greatly influence the chemical properties of the resulting ZIFs. Three different functional groups are available depending on the ZIFs used: the aldehyde group in ZIF-90 and Co-ZIF-90, the methyl group in ZIF-8 and ZIF-67, and the trifluoromethyl group in Zn(ImCF_3_)_2_. Previous studies report a much higher polarity of the framework introduced by the aldehyde group in ZIF-90 compared to the nonpolar methyl group in ZIF-8.^70^ As a result, adsorption of guest molecules of different polarity should occur at different relative pressures or guest contents in the atmosphere for the polar and nonpolar ZIFs. Consequently, subsequent loading of the pores of both MOF types should be possible when ZIFs with both functional groups are present in one system.

**Figure 6:**
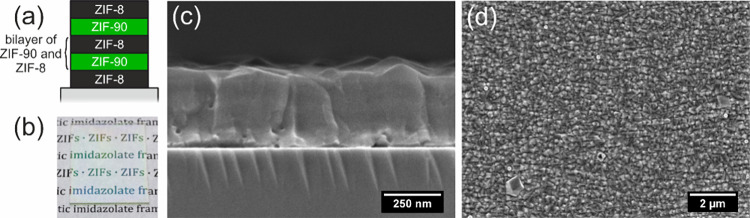
(a) Scheme of the model system used for adsorption studies, consisting of one seeding layer of ZIF-8 and two bilayers of ZIF-90 and ZIF-8. (b) Photograph of the thin film stack on a glass substrate, showing its reflective behavior. (c) Cross-section and (d) top-view SEM images of the synthesized model stack. The film stack for SEM was synthesized on a silicon substrate.

Therefore, we chose ZIF-8 and ZIF-90 as a Bragg-stack model system for guest adsorption studies with water, methanol and ethanol, creating a switchable photonic crystal. Another reason why we chose this system is that ZIF-8 and ZIF-90 show hardly any absorption in the visible range, which is an advantage for optical applications. We prepared alternating stacks of these two ZIFs, starting with a ZIF-8 seeding layer, followed by two bilayers of ZIF-90 and ZIF-8. The model system is built of five layers in total (Figure 6a). The film on glass, shown as a photography in Figure 6b is clear and transparent and has no visible absorption features. In the angle we chose for the photography, the reflective behavior of the film can be seen. The antireflective behavior was confirmed with UV–vis reflectance measurements, shown in Figure S11. In line with previous examples, SEM measurements (Figure 6c and d) confirm the successful fabrication of crack free and homogeneous thin films. A comparison of the experimental and fitted data for Ψ and Δ and the refractive indices of both ZIFs obtained from the fit are shown in ESI Figure S12. A high quality of the ellipsometry fit of this model system can be stated, including a good agreement of the refractive index dispersions for ZIF-8 and ZIF-90 already shown in Figure 3b.

An adsorption study for the ZIF-8/ZIF-90 stack was conducted, using two optical methods, UV–vis spectroscopy and ellipsometry. Methanol was selected as guest molecule for this study. To investigate the influence of the polarity of guest molecules, additional experiments with water (more polar) and ethanol (less polar) were performed and can be found in the ESI, Figures S13 and S14. All experiments were carried out on ZIF-8/ZIF-90 stacks or on powder samples of pure ZIF-8 and ZIF-90 for comparison.

Static vapor sorption measurements were carried out on ZIF-8 and ZIF-90 powder samples to determine the approximate relative pressures for the adsorption of the previously mentioned guest molecules. The XRD patterns of the powders are shown in ESI Figure S13 and the static vapor sorption isotherms for water and ethanol are shown in Figures S15 and S16.

**Figure 7:**
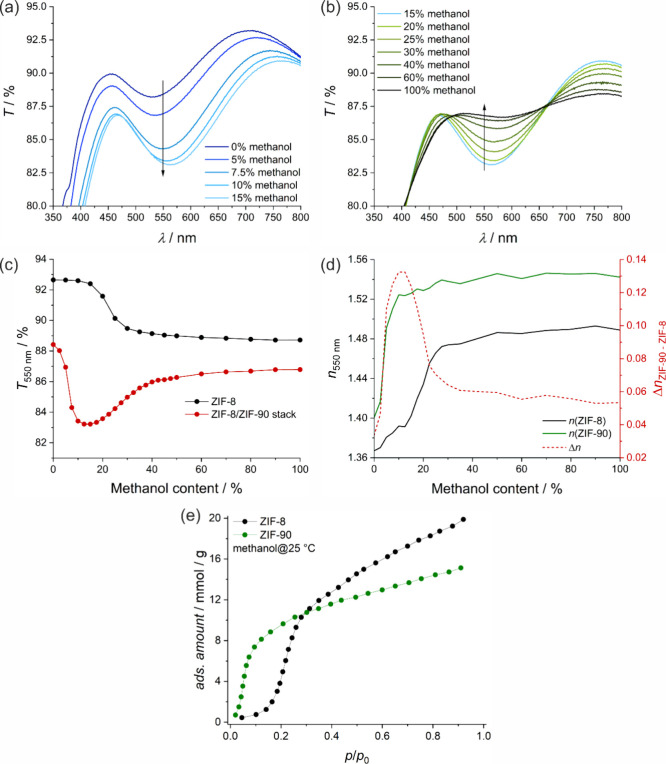
(a) and (b) show UV–vis transmission spectra measured through a glass slide coated with a ZIF-8/ZIF-90 stack (one seeding layer of ZIF-8 and two bilayers of ZIF-90 and ZIF-8) in gas atmospheres with different methanol concentration. The methanol concentration is varied between (a) 0 and 15% and (b) 15% and 100%, respectively. The evolution of the transmission values at 550 nm depending on the methanol concentration that results from (a) and (b) is shown in (c), red graph. It is compared to an analogous measurement that was performed on an ZIF-8-coated glass slide with five growth cycles (c), black graph. (d) Methanol concentration-dependent refractive indices of ZIF-8 (in black) and ZIF-90 (in green) and the refractive index difference (red dashed line). The measurements in (d) were performed on a coated silicon wafer. (e) Vapor sorption graphs of ZIF-8 and ZIF-90 powder as reference for the affinity to methanol.

The UV–vis transmission spectra of the ZIF-8/ZIF-90 stack measured in atmospheres with different methanol concentration are shown in Figure 7a-c. The measurements were performed using a home-built measuring cell with quartz glass windows and gas inlet and outlet, as described in detail in the ESI (Figure S14 and explanatory text). For the sake of clarity, the spectra are divided into two ranges: 0 to 15% (Figure 7a) and 15 to 100% of methanol (Figure 7b).

In the first range (up to 15% methanol), a decrease in transmission is observed over the entire spectral range, with the greatest decrease occurring around 550 nm (Figure 7a). In the second range (above 15% methanol), an increase in transmission around 550 nm is observed, while transmission below 490 nm and above 660 nm continues to decrease.

Figure 7c shows the evolution of the transmission at 550 nm for the ZIF-8/ZIF-90 stack (red curve) based on the data from Figure 7a and b. For comparison, the transmission of a similar ZIF-8 thin film at the same wavelength is also shown. The transmission of the ZIF-8/ZIF-90 stack decreases with increasing methanol content up to 15%. This decrease can be attributed to methanol adsorption in ZIF-90, as also observed in the powder sorption isotherm at low relative methanol pressure in Figure 7e. Methanol adsorption increases the refractive index of ZIF-90 (see Figure 7d, green curve), because methanol molecules fill the porous structure and contribute to the overall polarizability of the material. This results in an increased refractive index difference Δ*n* between the ZIF-90 and the ZIF-8 layers (Figure 7d, red curve). According to Fresnel’s law, a greater refractive index difference between layers results in increased reflection at the interfaces. When light encounters a boundary between two media with different refractive indices, a portion of the light is reflected while the rest is transmitted, thereby explaining the observed decrease in transmission through the ZIF-8/ZIF-90 stack.

The transmission of the pure ZIF-8 thin film remains stable up to a methanol concentration of about 15%. As the methanol concentration increases further, the transmission decreases between 15 and 40%, which can be explained by an increase in the refractive index due to methanol adsorption in ZIF-8 (Figure 7d). When considering the ZIF-8/ZIF-90 stack, this adsorption results in an increase in transmission between 15 and 40%. This occurs because methanol adsorption increases the refractive index of ZIF-8 (Figure 7d, black curve), while the refractive index of ZIF-90 remains relatively unchanged. This reduced refractive index difference Δ*n* between the layers causes the transmission to increase again. In general, the changing refractive index difference between the two layers influences transmission: A larger refractive index difference reduces transmission, while a smaller difference increases it.

These observations are consistent with the results of the methanol vapor sorption on powder samples, where methanol uptake is also observed at similar relative methanol pressures for ZIF-8 powder (see Figure 7e). Methanol adsorption occurs in both frameworks, ZIF-90 and ZIF-8. In ZIF-90, adsorption takes place at low relative pressures, resulting in a Type I isotherm, while in ZIF-8, the main adsorption step occurs at a relative pressure of 0.2.

A notable observation is the exclusive adsorption of water in ZIF-90 layers due to the high hydrophobicity of ZIF-8, which repels water, while the polar nature of ZIF-90 facilitates adsorption. The placement of ZIF-8 as the top layer does not hinder water adsorption in the underlying ZIF-90 layer, which we attribute to the small kinetic diameter of water molecules, allowing them to pass through the ZIF-8 pores.

As mentioned earlier, similar experiments were performed using water (more polar) and ethanol (less polar) as guest molecules. Vapor sorption graphs, UV–vis transmission spectra and derived content-dependent transmission diagrams are shown in ESI Figure S15 (for water) and Figure S16 (for ethanol). In the vapor sorption, the more polar water is adsorbed in the ZIF-90 with a sharp edge at 0.4 relative pressure, while ZIF-8, due to its hydrophobicity, adsorbs almost no water. Ethanol is adsorbed by both ZIF-90 and ZIF-8, with adsorption taking place at a lower relative pressure of around 0.05. This behavior is also reflected in the UV–vis measurements. The ZIF-8/ZIF-90 layers show a drastic transmission decrease at around 30% water content, resulting from the exclusive filling of the ZIF-90 layer. As ethanol is adsorbed in both layers at concentrations around 5%, the minimum of transmission is shifted to lower ethanol contents, compared to methanol.

In addition to the complete UV–vis spectra, time-dependent measurements at a fixed wavelength of 550 nm were conducted. During these measurements, the vapor concentration was alternated between 0% and 100% at 30-s intervals over a period of 30 min to assess the stability of the signals. The corresponding data are shown in Figures S17 to S19 in the ESI.

For all three vapors, the signals remain stable over the entire measurement period. Additionally, the adsorption of ethanol and methanol occurs in two distinct steps, whereas water adsorption proceeds in a single step. These results are consistent with those from the previous UV–vis, ellipsometry and vapor sorption measurements.

An SEM image of the sample after the measurement, shown in Figure S20 in the ESI, confirms that the film remained intact after various measurement steps.

This investigation demonstrates, that the polarity and sorption behavior of ZIF derivatives are strongly influenced by the linker molecule. These properties are successfully transferred from the powder form to the novel thin film stacks. This allows the sequential fine-tuning of the refractive index in individual layers or both layers, depending on the guest concentration. A substantial difference in the refractive indices between the layers induces a notable change in the optical transmission properties of the film stacks. This effect can be used to control or modulate the light transmission through these thin films.

## 4 Conclusions

We have extended a previously reported ZIF-8 seeding layer approach for synthesizing Co-ZIF-90 and present three additional ZIFs that can be grown as thin films of optical quality using ZIF-8 as a seeding layer: ZIF-67, ZIF-90 and Zn(ImCF_3_)_2_. The synthesis of these thin films is rapid, in less than 1 h or slightly longer for thicker films, using a cycle based process. We characterized their optical properties, including transmission of ZIF-coated glass slides and refractive index via ellipsometry on silicon wafers. The seeding layer approach effectively grows layered ZIF stacks in a desired and distinct sequence. Many combinations are possible, starting with ZIF-8 on glass, with each layer crystallizing in optical quality. The resulting materials were analyzed using ellipsometry, EDX mapping and XPS depth profiling.

This method allows for the construction of a database of ZIF derivatives for various Bragg-stack combinations. Among these, we selected alternating stacks of ZIF-8 and ZIF-90 for further studies. Functional groups at the 2-position of the imidazole derivative, specifically the nonpolar methyl group in ZIF-8 and the polar aldehyde group in ZIF-90, control guest molecule adsorption. In layered ZIF-8/ZIF-90 systems, this results in preferential and sometimes exclusive adsorption in one of the layers. UV–vis transmission experiments and ellipsometry measurements under controlled vapor conditions provided insights into adsorption of water, methanol and ethanol, revealing an optical response. The transmittance change in the ZIF-8/ZIF-90 thin film stack is attributed to refractive index differences of the two MOFs depending on their guest content.

The ZIF-8 seeding layer approach represents a novel method for the fast and mild synthesis of high-quality ZIF coatings with enhanced optical properties. Unlike previous MOF-only systems, our synthesized thin films utilize the intergrowth of various ZIFs in **sod** topology. This approach creates a versatile library of layered systems but also holds potential for extension to other ZIF derivatives. The design of this system allows fine-tuning of optical properties through strategic choices of linkers, metals, guest molecule polarity and concentration.

## Supporting Information Available

Additional information about the thin film synthesis, the evaluation of the ellipsometry data and further SEM images of the MOF thin films are provided. Material for the characterization of layered ZIF stacks and ZIF powder samples are shown. Finally, details about the guest loading experiments are provided (PDF)

## Dedication

^†^ Dedicated to the memory of Prof. Dr. Peter Behrens, who sadly passed away on January 13, 2023.
