# Supplementary material for: Adsorption-Induced Optical Modulation in ZIF Thin Film Stacks with Distinct Order for Photonic Crystal Applications
Source: ACS Appl Mater Interfaces. 2025 May 1;17(19):28830–40. doi: 10.1021/acsami.5c02529 (PMC12086767; doi:10.1021/acsami.5c02529)
Supplement: Supplementary file 1 — am5c02529_si_001.pdf [file am5c02529_si_001.pdf]

Electronical supporting material for

Adsorption-induced optical modulation in ZIF thin film stacks with distinct order for photonic crystal applications

Nils Christian Keppler,[a,b] Lukas Steinbach,[a,b] Johanna Fricke,[a] Adrian Hannebauer,[a] Erik Rohloff,[a] Andreas Schaate,*[a,b] Peter Behrens[a,b]

[a] Institute of Inorganic Chemistry, Leibniz University Hannover, Hannover, Germany

[b] Cluster of Excellence PhoenixD (Photonics, Optics and Engineering – Innovation Across Disciplines), Leibniz University Hannover, Hannover, Germany

*Corresponding author, andreas.schaate@acb.uni-hannover.de

Table of contents

Adsorption-induced optical modulation in ZIF thin film stacks with distinct order for photonic crystal applications 1

1. Additional information on the thin film synthesis 2

2. Evaluation of ellipsometry data 1

3. Additional SEM images 3

4. Characterization of mixed ZIF stacks 5

    4.1Thin film stack of ZIF-8/ZIF-67/ZIF-90/Co-ZIF-90 5

    4.2 XPS spectra of thin film stack of ZIF-8/Co-ZIF-90/ZIF-90 7

    4.3 XPS spectra of thin film stack of ZIF-8/ZIF-67/ZIF-90/Co-ZIF-90 9

    4.3 Thin film stack of ZIF-8 and ZIF-90 with alternating layers 11

5. Characterization of ZIF powder samples of ZIF-8 and ZIF-90 12

6. Supporting material for the guest loading experiments (UV-Vis and ellipsometry) 13

7. Cyclic reversibility and stability tests for ZIF-8 and ZIF-90 stacks 16

8. References 18

1. Additional information on the thin film synthesis

The precursors, their concentrations and the solvents used for the synthesis of the various different ZIF thin films are summarized in Tab. S1. All syntheses were performed at room temperature (approximately 18 °C) with a synthesis time of 30 min per deposition cycle. A seeding layer of ZIF-8 (one deposition cycle) is necessary for the syntheses of ZIF-67, $Zn(ImCF_3)_2$, ZIF-90 and Co-ZIF-90.

Tab. S1: Precursors, concentrations and solvents for the syntheses of the different ZIF thin films.

ZIF	Precursors	c/ mM	Solvent
ZIF-8	$Zn(NO_3)_2 \cdot 6\,H_2O$	25	Methanol
	2-methylimidazole (HmIm)	50	Methanol
ZIF-67	$Co(NO_3)_2 \cdot 6\,H_2O$	25	Methanol
	2-methylimidazole (HmIm)	50	Methanol
$Zn(ImCF_3)_2$	$Zn(NO_3)_2 \cdot 6\,H_2O$ + $Zn(OAc)_2 \cdot 2\,H_2O$ (1:1)	10	Methanol
	2-(trifluoromethyl)-imidazole ($HImCF_3$)	20	DMF
ZIF-90	$Zn(NO_3)_2 \cdot 6\,H_2O$	25	Methanol
	Imidazole-2-carbaldehyde (HImCA)	100	DMF
Co-ZIF-90	$Co(NO_3)_2 \cdot 6\,H_2O$ + $Co(OAc)_2 \cdot 4\,H_2O$ (2:1)	12.5	Methanol
	Imidazole-2-carbaldehyde (HImCA)	50	DMF

2. Evaluation of ellipsometry data

Details about the ellipsometry fitting strategy for the different ZIF thin films are given in Tab. S2. A Tauc-Lorentz model was used for the fitting of ZIF thin films that absorb in the range of the visible light spectrum (ZIF-67 and Co-ZIF-90, both exhibiting a purple color).

A Cauchy model was used to describe ZIF films that are not absorbing light from the visible light spectrum (ZIF-8 and $Zn(ImCF_3)_2$). ZIF-90, which has a slightly yellowish color and a band gap near the visible range, was also better described using Tauc-Lorentz model.

For the Tauc-Lorentz fits, up to three oscillators were used:

- One oscillator to describe the band gap adsorption in the UV range
- Two oscillators to account for absorption in the visible range (for ZIF-67 and Co-ZIF-90)

Tab. S2: Details about the ellipsometry fits.

Layer	Parameter	Value
air	Fixed n and k values	$n = 1, k = 0$
Roughness layer	Layer type	Effective medium layer
		Top layer: air
		Bottom layer: top MOF layer
	Fraction of inclusion	0.5
	Thickness	Free
ZIF-8 layer	Layer type	Cauchy-Layer
	N0	Free* or 1.336**
	N1	Free* or 52.0**
	N2, K0, K1, K2	0
	Thickness	Free
$Zn(ImCF_3)_2$	Layer type	Cauchy-Layer
	N0	Free
	N1	Free
	N2, K0, K1, K2	0
	Thickness	Free
ZIF-90	Layer type	Tauc-Lorentz-Layer
	Number of oscillators	1
	E_0, E_g, A, C	Free
	Thickness	Free
Co-ZIF-90 layer	Layer type	Tauc-Lorentz-Layer
	Number of oscillators	3
	E_0, E_g, A, C	Free
	Thickness	Free
ZIF-67	Layer type	Tauc-Lorentz-Layer
	Number of oscillators	3
	E_0, E_g, A, C	Free
	Thickness	Free

*only ZIF-8 on silicon *without* additional other MOF layers

**for a system *with* additional thin films (ZIF-8 as the seeding layer) to reduce the number of fit parameters; the values $N0 = 1.336$ and $N1 = 52.0$ have been evaluated from more than ten measurements on different samples.

3. Additional SEM images

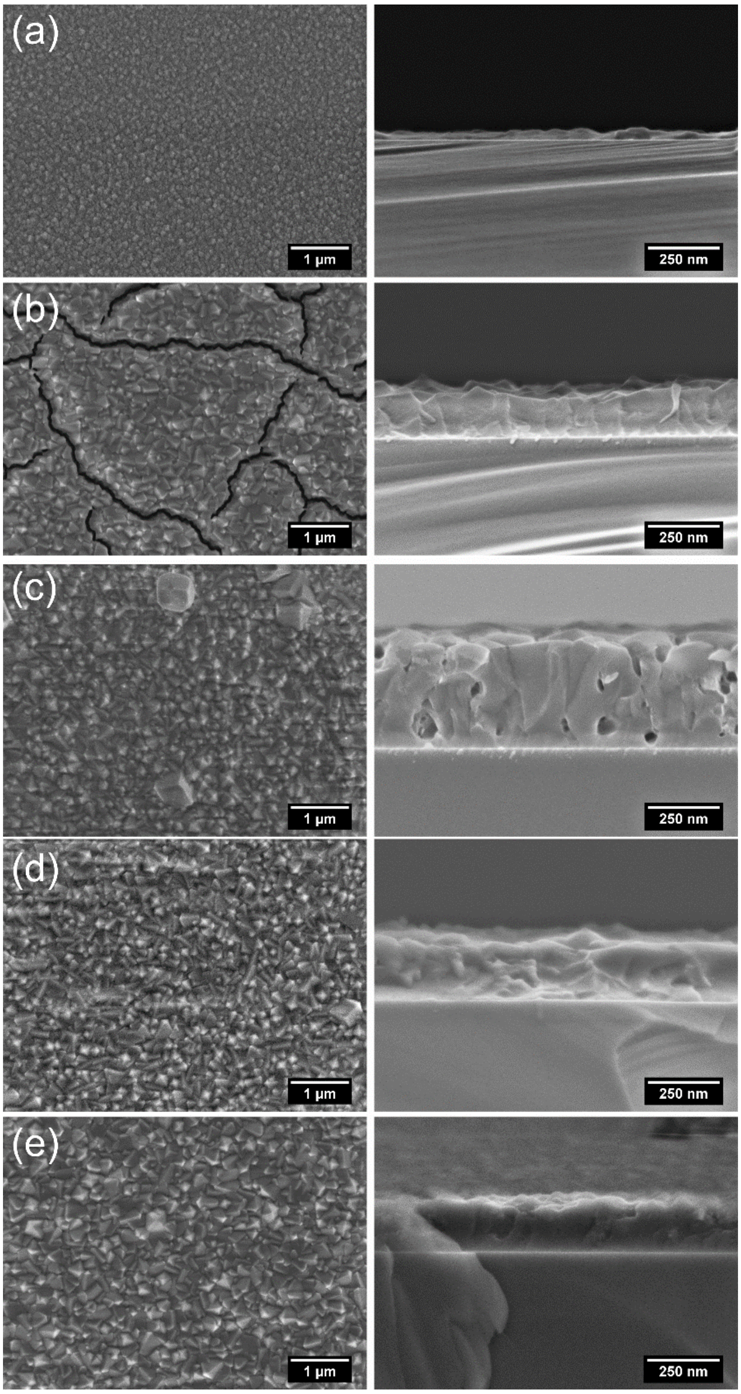

Fig. S1: Top-view and cross-sectional SEM images of all ZIF thin films syntheiszed on silicon substrates. The images show (a) the ZIF-8 seeding layer, (b) ZIF-90, (c) $Zn(ImCF_3)_2$, (d) ZIF-67, and (e) Co-ZIF-90, each deposited in two deposition cycles on a ZIF-8 seeding layer.

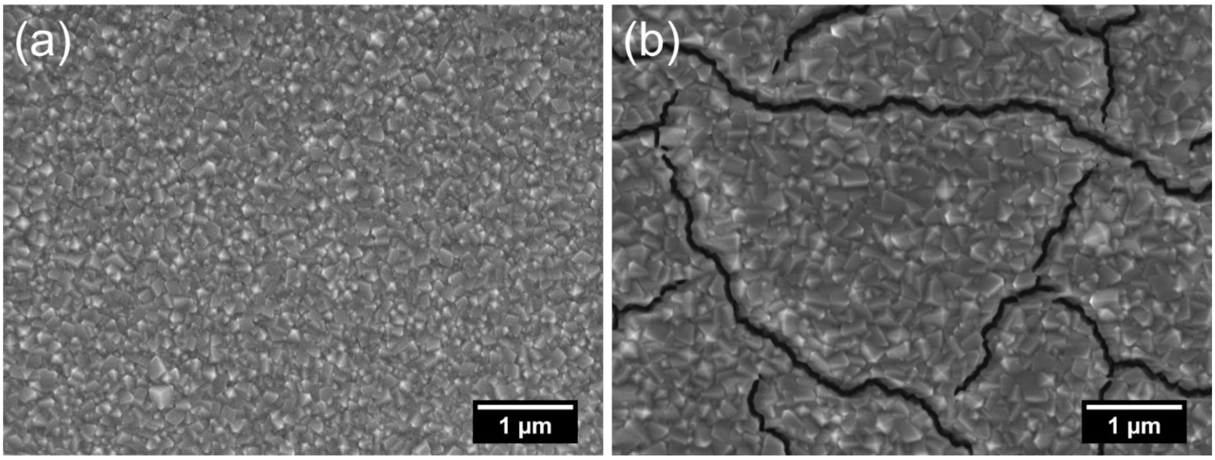

Fig. S2: Comparison of SEM images of ZIF-90-on-ZIF-8 coatings with (a) one and (b) two deposition cycles applied for the ZIF-90 top layer.

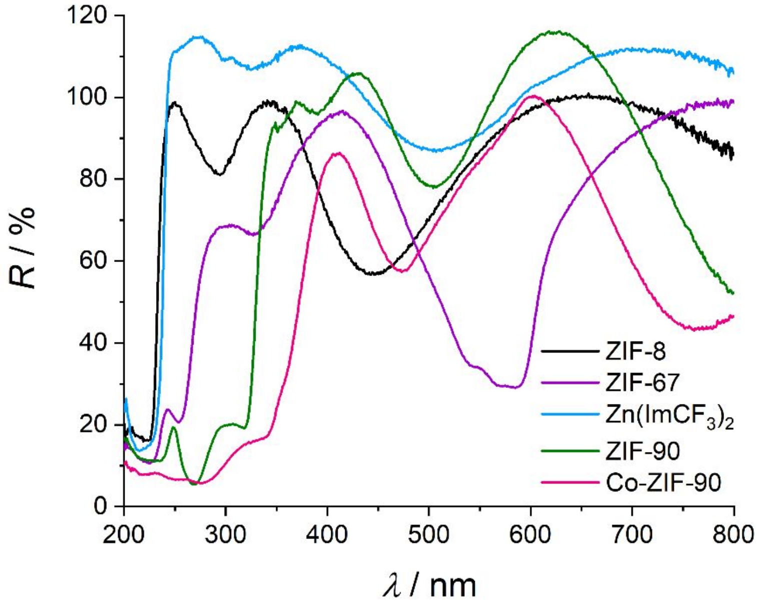

Fig. S3: Reflectance spectra of thin films on a silicon wafer with (111) orientation. A bare silicon wafer was used as background reference to improve the visibility of anti-reflective behavior. Each ZIF film consists of one ZIF-8 seeding layer followed by two consecutive layers, as shown in the legend.

4. Characterization of mixed ZIF stacks

4.1 Thin film stack of ZIF-8/ZIF-67/ZIF-90/Co-ZIF-90

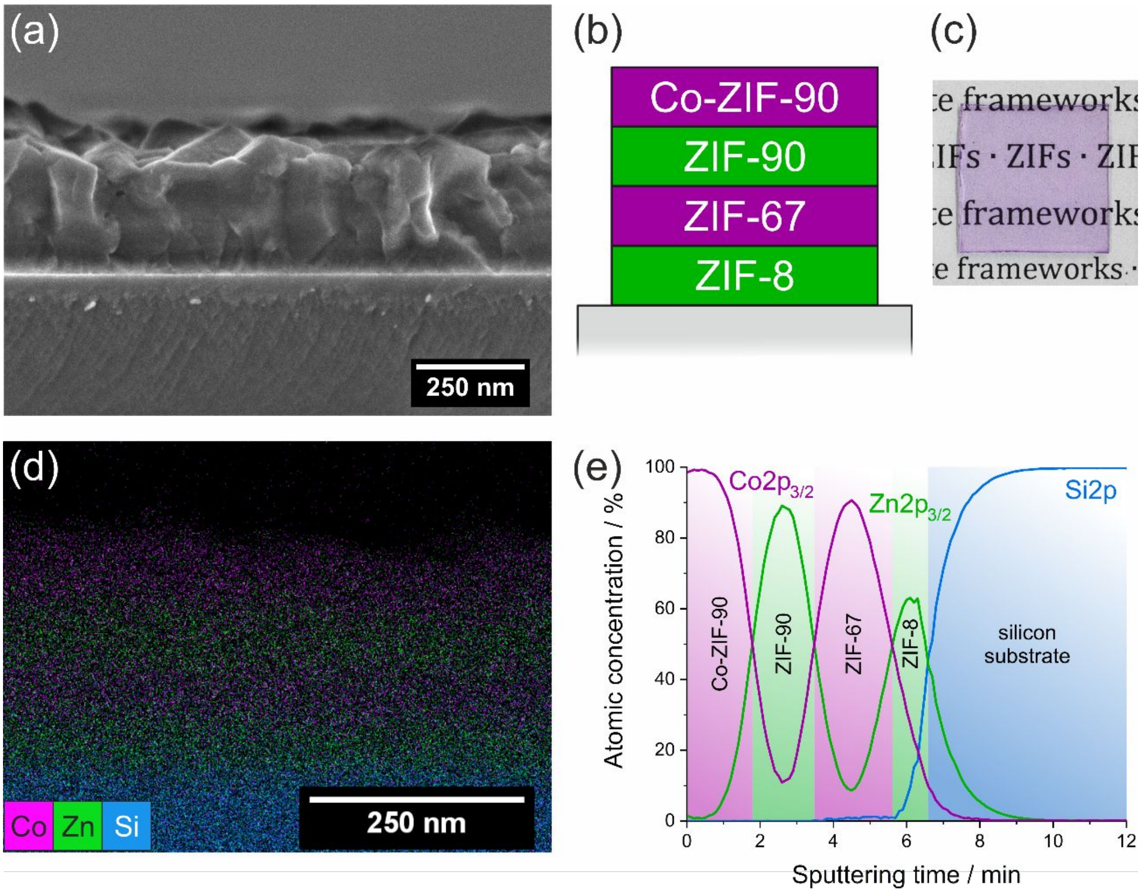

Fig. S4: (a) Cross-sectional SEM image of the four-layer stack consisting of ZIF-8/ZIF-67/ZIF-90/Co-ZIF-90, fabricated on a silicon substrate. (b) Schematic representation of the layer sequence. (c) Photograph of the thin film stack coated on a glass substrate. (d) EDX mapping of a cross-section and (e) XPS depth profile measurement, both performed on samples fabricated on silicon substrates.

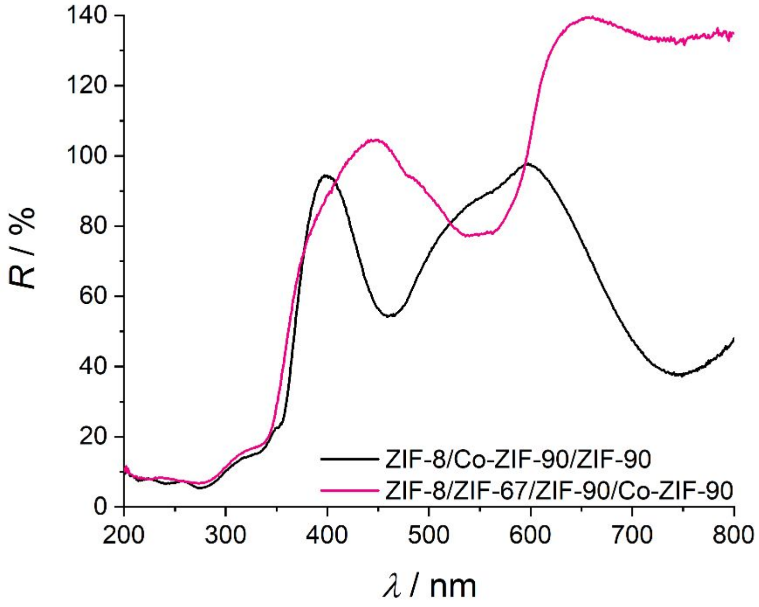

Fig. S5: Reflectance spectra of three- and four-layer ZIF thin films stacks measured on a silicon wafer with (111) orientation. A bare silicon wafer was used as background to improve the visibility of anti-reflective behavior.

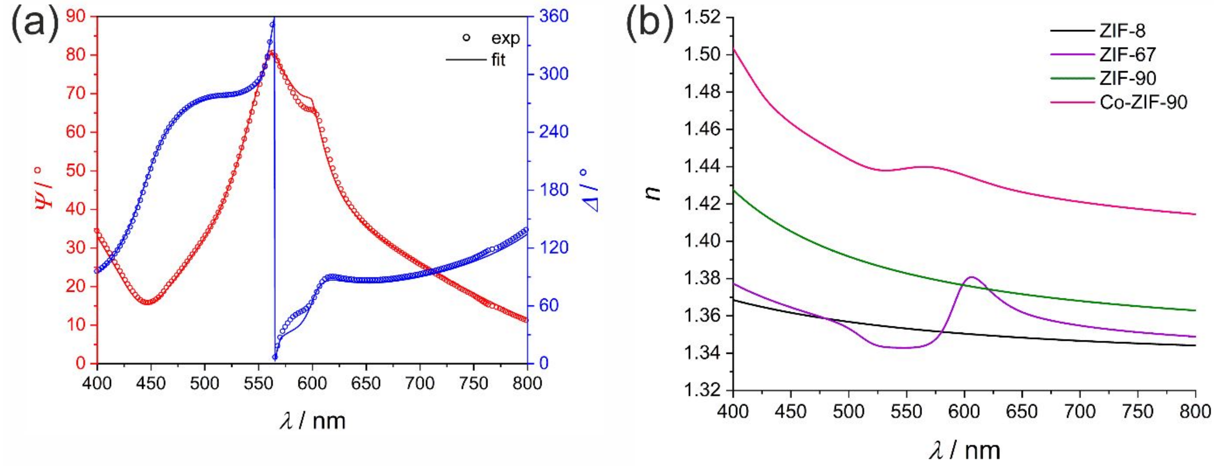

Fig. S6: Ellipsometry result for the ZIF-8/ZIF-67/ZIF-90/Co-ZIF-90 stack on silicon. The result of the fit - experimental (circles) and fitted (line) curves for Ψ and Δ - are shown in (a). All resulting refractive index dispersions are presented in (b).

4.2 XPS spectra of thin film stack of ZIF-8/Co-ZIF-90/ZIF-90

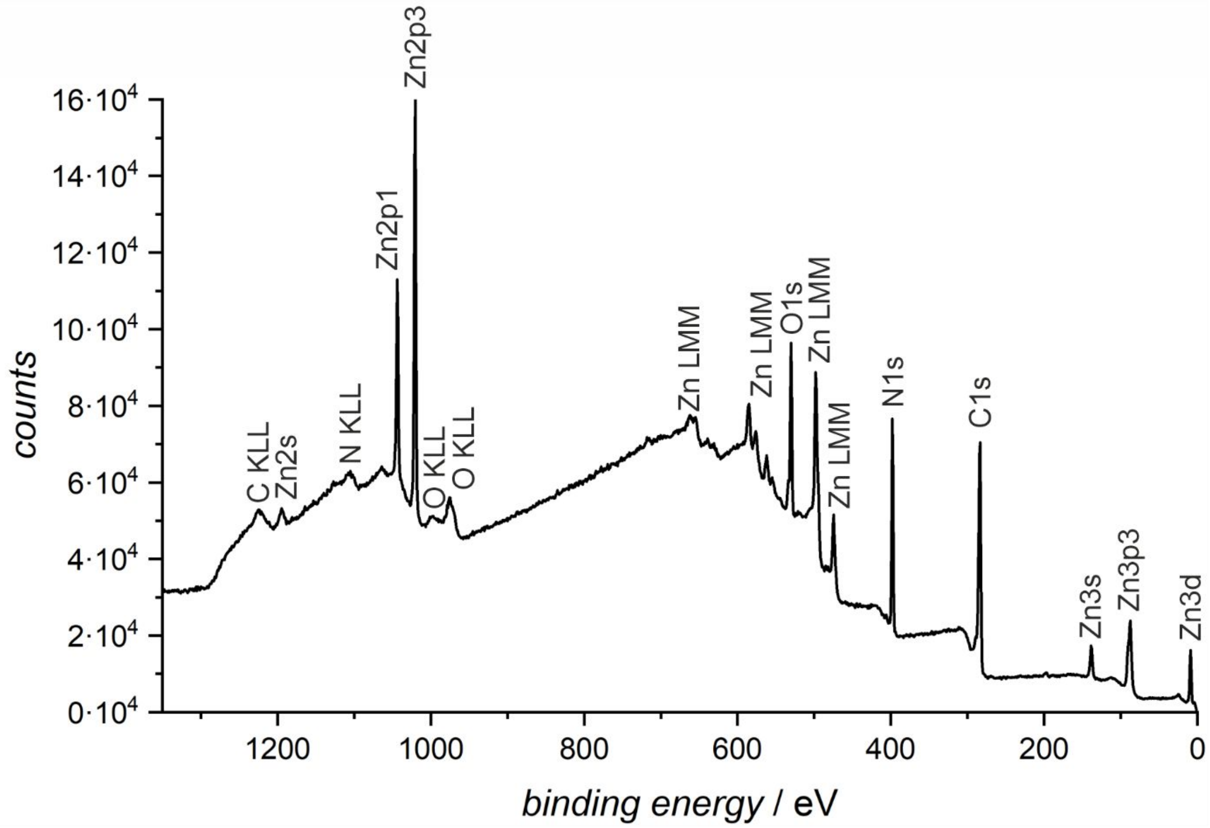

Fig. S7: XPS survey spectrum of the ZIF-8/Co-ZIF-90/ZIF-90 film stack recorded before the sputtering process. Characteristic peaks are labelled accordingly.

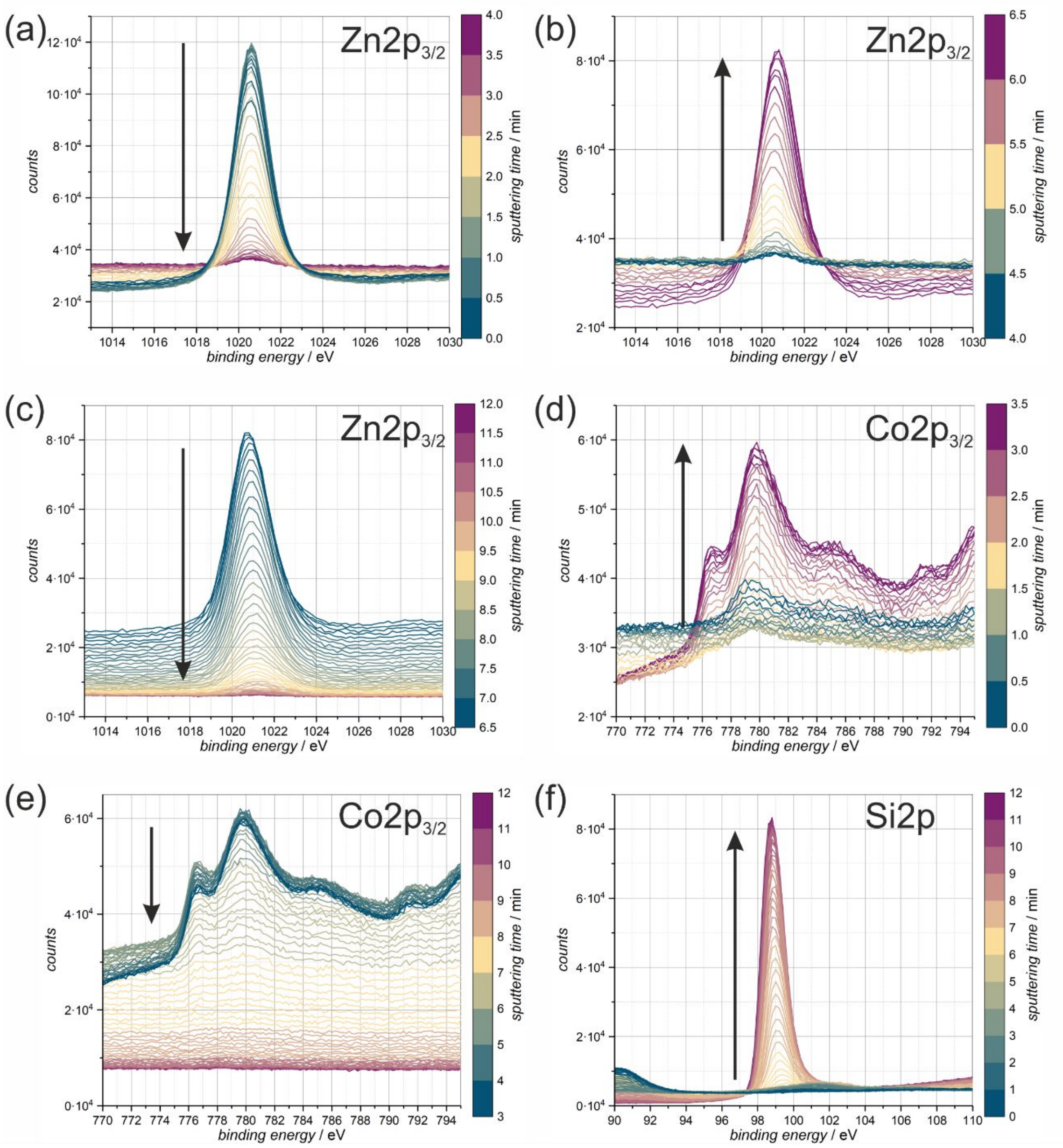

Fig. S8: XPS spectra of the sputtering experiments on the ZIF-8/Co-ZIF-90/ZIF-90 film stack. (a) to (c) show the evolution of the zinc signal with increasing sputtering time, (d) and (e) show the cobalt signal and (f) shows the silicon signal. Arrows indicate the signal progression within each spectrum. The XPS narrow scans presented here depict the evolution of key elemental signals (Zn 2p3/2, Co 2p3/2, Si 2p) as a function of sputtering time and provide qualitative insight into the layered composition of the thin films. In both layered systems, the depth profiles clearly reflect the intended stacking of zinc- and cobalt-containing ZIFs, supporting the conclusion that no noticeable cation exchange occurs during film growth.

4.3 XPS spectra of thin film stack of ZIF-8/ZIF-67/ZIF-90/Co-ZIF-90

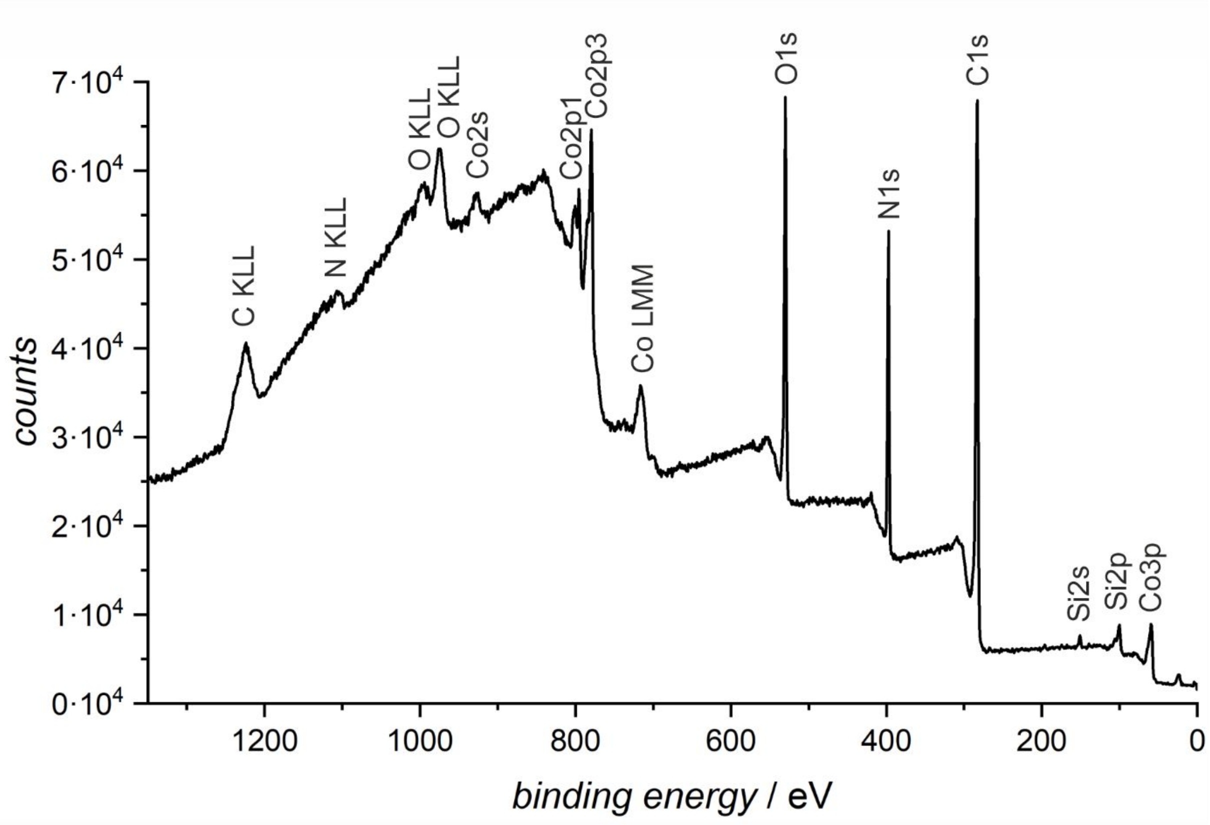

Fig. S9: XPS survey spectrum of the ZIF-8/ZIF-67/ZIF-90/Co-ZIF-90 film stack recorded before sputtering process. Characteristic peaks are labelled accordingly.

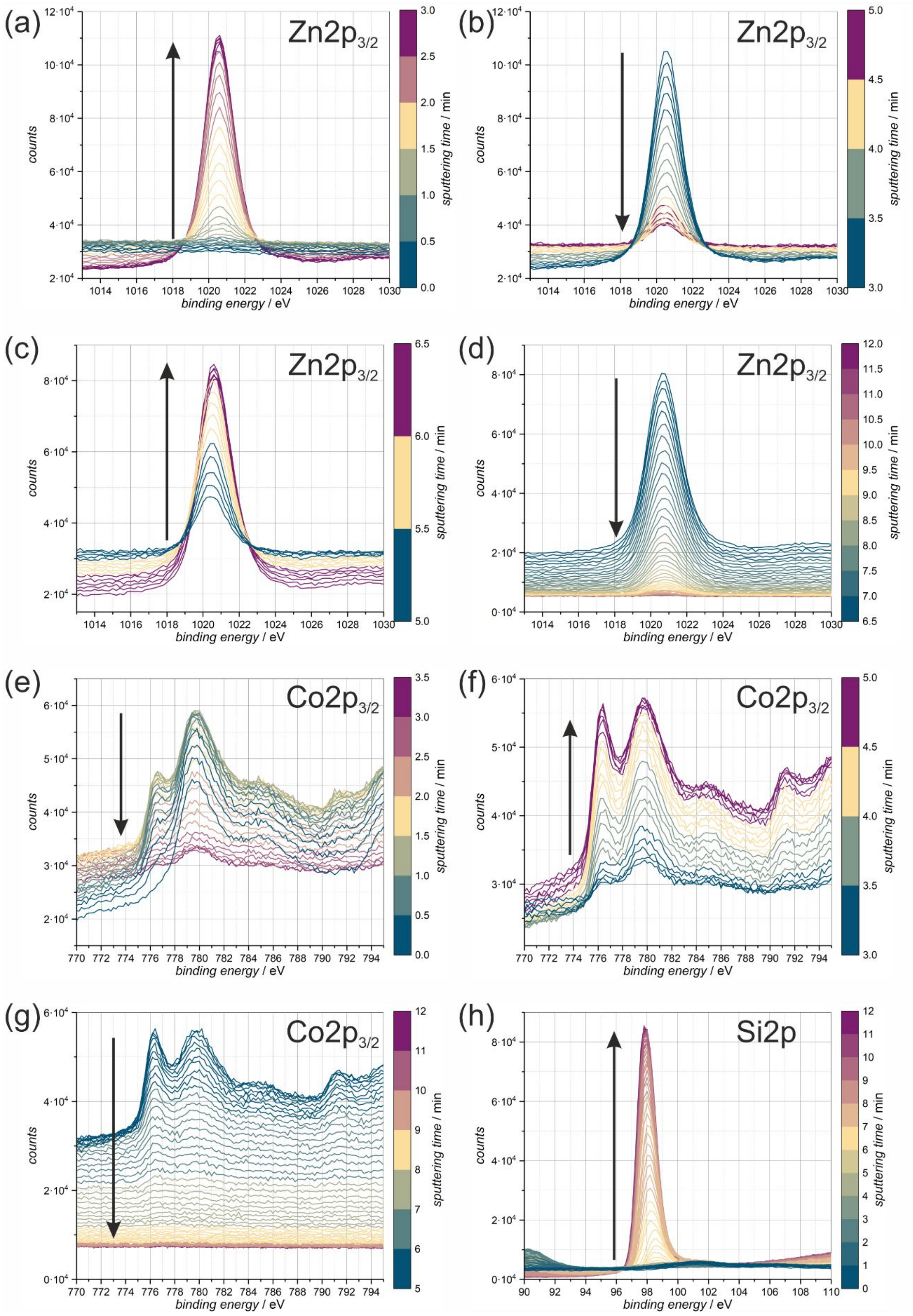

Fig. S10: XPS spectra of the sputtering experiments on the ZIF-8, ZIF-67, ZIF-90 and Co-ZIF-90 film stack. (a) to (d) show evolution of the zinc signal with increasing sputtering time, (e) to (g) show the evolution of the cobalt signal and (h) shows the silicon signal. Arrows indicate the signal progression within each spectrum. The XPS narrow scans presented here show the evolution of key elemental signals (Zn 2p3/2, Co 2p3/2, Si 2p) as a function of sputtering time and provide qualitative insight into the layered composition of the thin films. In both layered systems, the depth profiles clearly reflect the intended stacking of zinc- and cobalt-containing ZIFs, supporting the conclusion that no noticeable cation exchange occurs during film growth.

4.3 Thin film stack of ZIF-8 and ZIF-90 with alternating layers

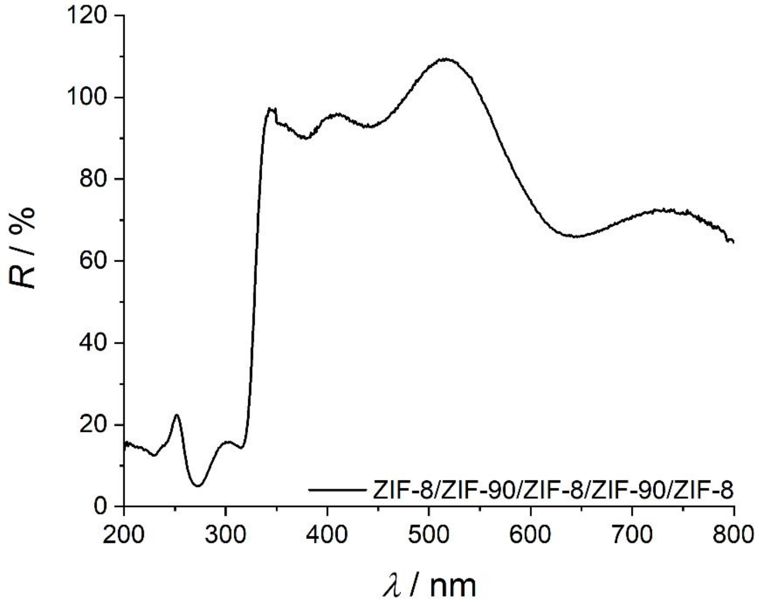

Fig. S11: Reflectance spectrum of the five-layer ZIF thin films stacks consisting of ZIF-8 and ZIF-90, measured on a silicon wafer with (111) orientation. A bare silicon wafer was used as background to improve the visibility of anti-reflective behavior.

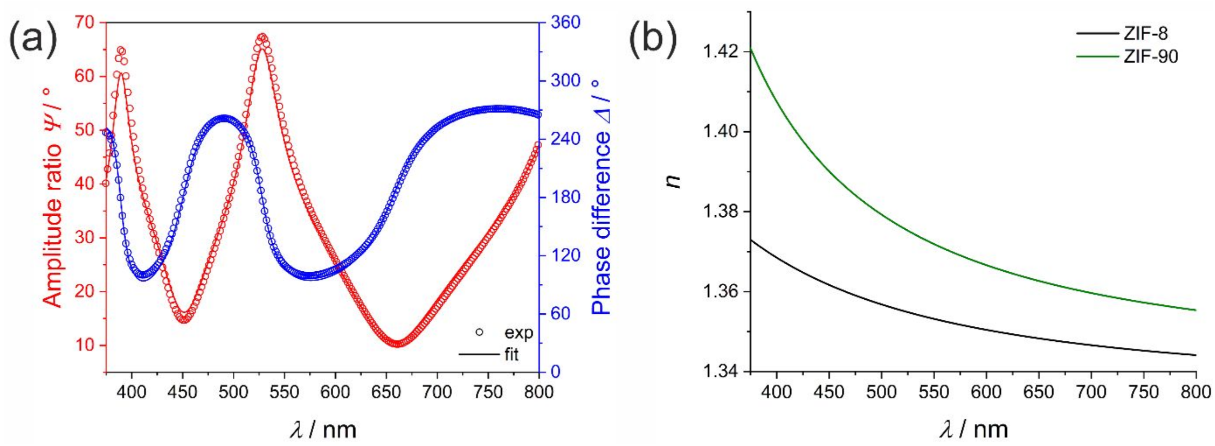

Fig. S12: Ellipsometry result for two bilayers of ZIF-8 and ZIF-90 with an additional ZIF-8 on silicon. The result of the fit - experimental (circles) and fitted (line) curves for *Ψ* and *Δ* - are shown in (a). All resulting refractive index dispersions are presented in (b).

5. Characterization of ZIF powder samples of ZIF-8 and ZIF-90

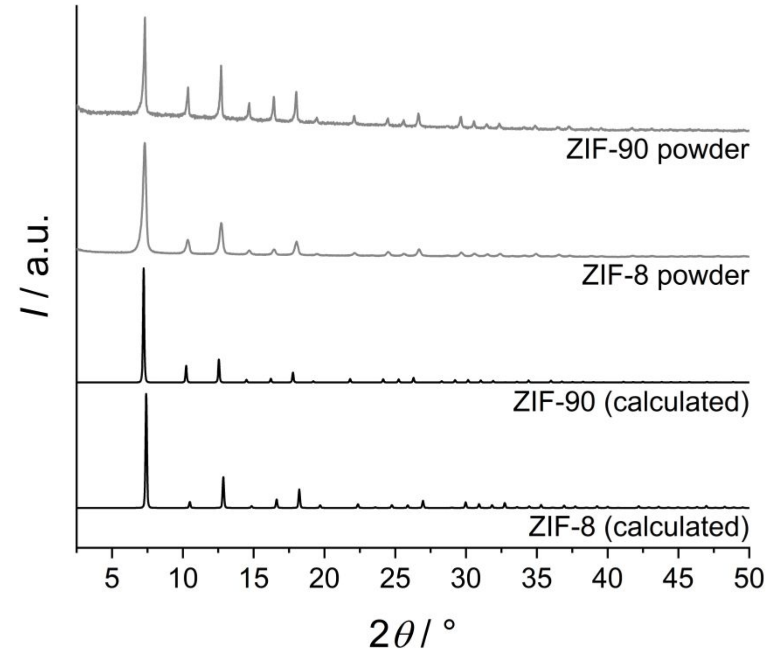

Fig. S13: XRD patterns of ZIF-8 and ZIF-90 powder samples (in grey). The calculated XRD pattern of both ZIFs are given as a reference (in black).[1,2] A good agreement of the reflections of both ZIFs with the calculated data can be seen.

6. Supporting material for the guest loading experiments (UV-Vis and ellipsometry)

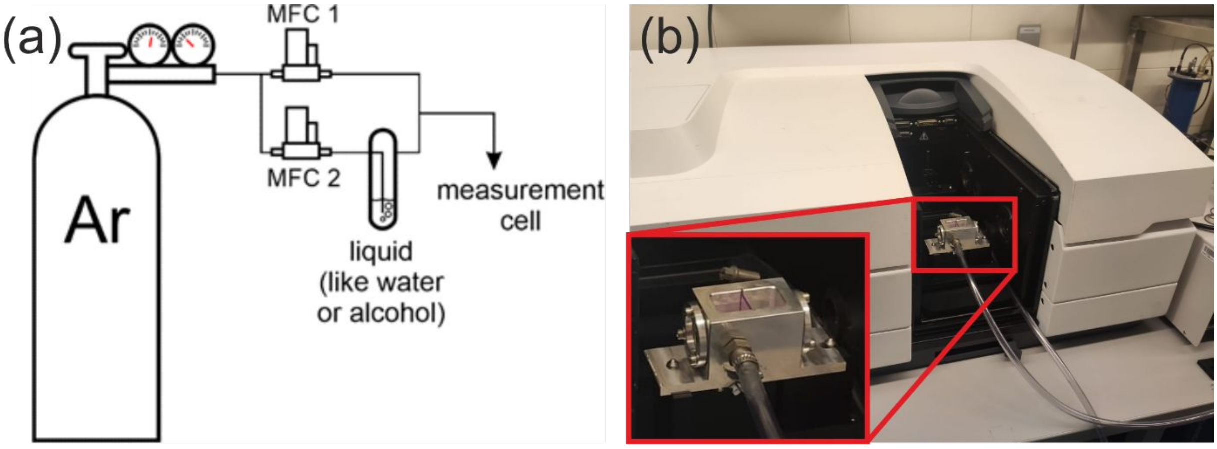

Fig. S14: Schematic setup for the optical measurements in controlled atmospheres using water, methanol or ethanol as guest (a). The setup was used for UV-Vis transmission experiments with the Cary 400 UV-Vis shown in (b) and for ellipsometry measurements.

We give a detailed description of the experiments in controlled guest atmospheres here. All UV-Vis experiments with controlled gas atmosphere described in the main text were carried out on glass slides with a coating of ZIF-8 (three deposition cycles) or a ZIF-8/ZIF-90 stack (2 bilayers + ZIF-8). The transmission through a ZIF-coated glass slide was measured in the wavelength range from 300 to 800 nm at different atmospheres. The setup for the control of the gas atmosphere is shown in Fig. S12b. Guest contents of the three guests (water, methanol or ethanol) were controlled using two mass-flow controllers – one for the guest flow (MFC 2) and one for the guest-free argon flow (MFC 1). Argon was used as the feed gas for both gas flows. The total gas flow was set to 1 L/min. 0% guest content was realized by opening only MFC 1 and 100% guest content by only opening MFC 2. Guest contents between 1% and 99% can be set by mixing the two gas flows with the desired composition, *e. g.* 0.95 L/min at MFC 1 and 0.05 L/min at MFC 2 to set the guest content to 5%.

Ellipsometry measurements in controlled atmosphere were carried out analogously but samples were prepared on silicon substrates. We used samples with a ZIF-90 layer (two deposition cycles) on a ZIF-8 seeding layer for these experiments to reduce the high number of fit parameters that would result from a ZIF stack with more layers (*e. g.* the above described ZIF-8/ZIF-90 stack with 2 bilayers + ZIF-8 top layer).

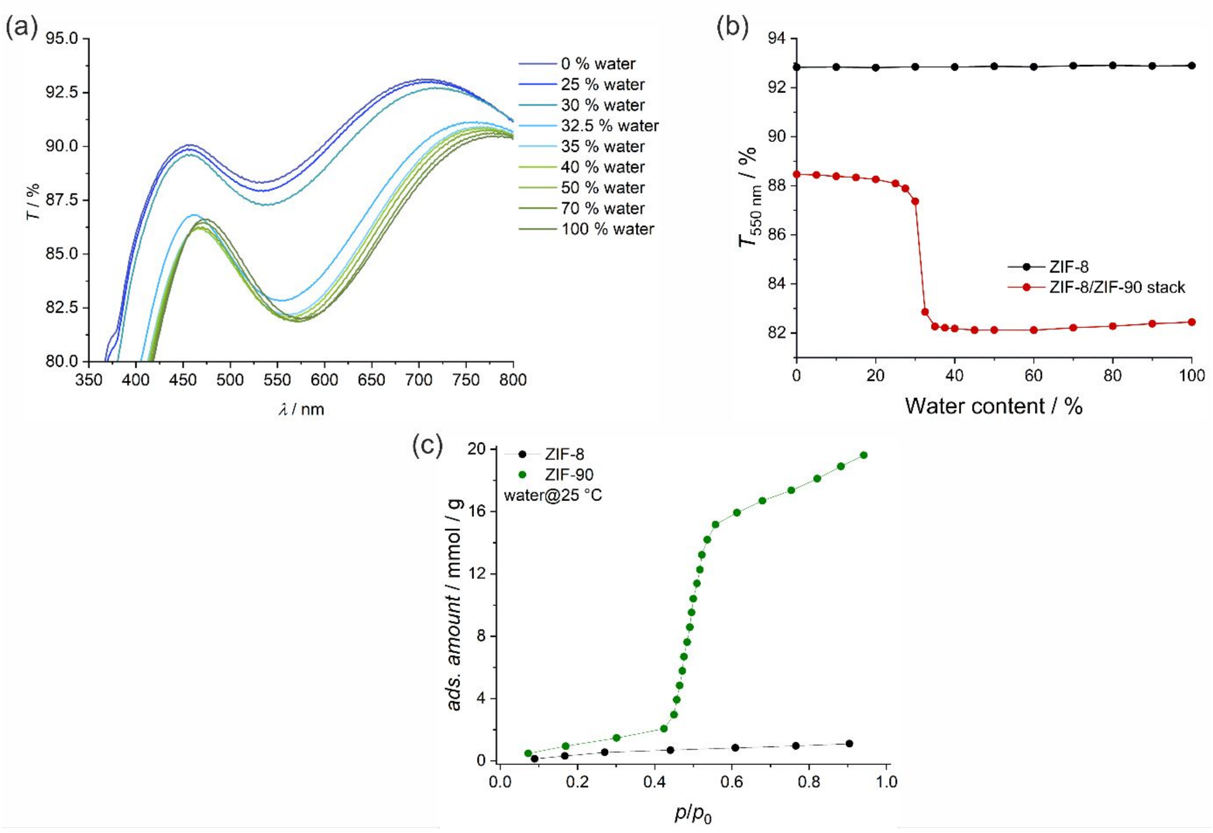

Fig. S15: UV-Vis transmission spectra measured through glass slides coated with a ZIF-8/ZIF-90 stack (one seeding layer of ZIF-8 two bilayers of ZIF-90 and ZIF-8) in a gas atmosphere with controlled water content. The water content is varied between 0 and 100 %.

Fig. S8 shows the results obtained from the water sorption study. In the vapor sorption measurement Fig. S8c, the ZIF-8 adsorbs nearly no water, due to its hydrophobic, non-polar characteristics, while ZIF-90 on the other hand show strong water adsorption over a relative pressure over 0.40. This is confirmed by UV-Vis measurements, Fig. 8Sa and b: The transmittance of a ZIF-8 coated glass slide does not change at different water contents. However, since water can be adsorbed in ZIF-90 due to the more polar aldehyde group, a transmission decrease can be observed for the ZIF-8/ZIF-90 stack around a water content of about 30%, visible in the single UV-Vis spectra, as well as in the derived graph for the transmission at 550 nm. Subsequently, the transmission reaches a plateau and does not increase again, since water adsorption cannot take place in ZIF-8. This is in good agreement with the vapor sorption measurements performed on ZIF-8 and ZIF-90 powder samples, see Fig. 8Sc. Similar results were also obtained on a coating of ZIF-8 and Co-ZIF-90.[30] The refractive index of Co-ZIF-90 with a comparable polarity to ZIF-90, shows an increase in refractive index at about 30% relative humidity caused by water adsorption in the framework.

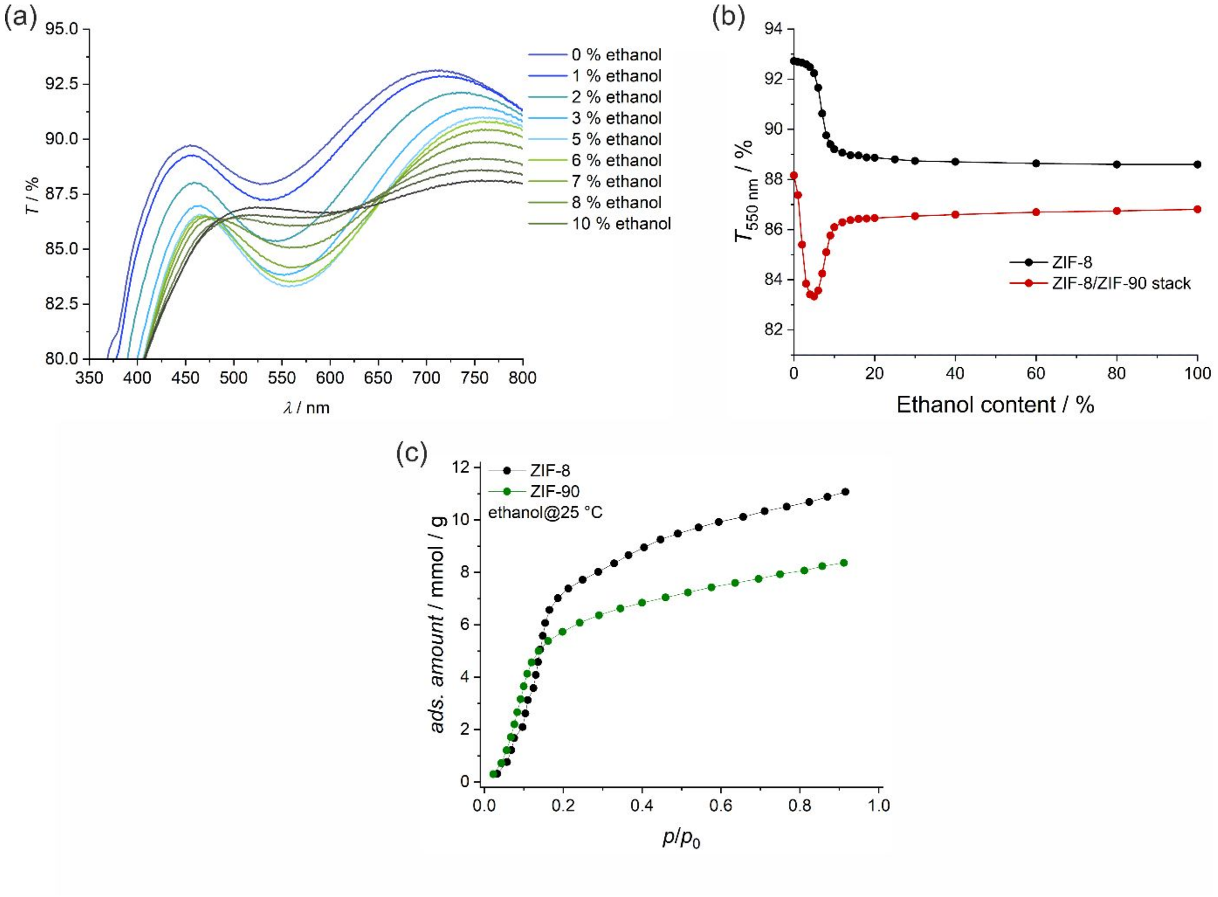

Fig. S16: UV-Vis transmission spectra measured through glass slides coated with a ZIF-8/ZIF-90 stack (2 bilayers + ZIF-8 top layer) in a gas atmosphere with variable ethanol content. The ethanol content is varied between 0 and 100%.

Fig. S9 shows the results obtained from the ethanol sorption study. In the vapor sorption measurement Fig. S9c, the ZIF-8 and ZIF-90 show a similar behavior, with the inset of adsorption around 0.05 relative pressure. For both materials the adsorption is reduced around 0.15 relative pressure. ZIF-8 shows a higher total uptake of ethanol, which can be attributed to its greater pore volume and increased accessibility. Furthermore, the transmission of the ZIF-8/ZIF-90-coated glass slide changes with increasing ethanol content as shown in Fig. 9a and b. Again, different ranges of adsorption can be defined: Between 0 and 5% of ethanol, there is a decrease in transmission caused by the adsorption of ethanol in ZIF-90. By further increasing the ethanol content between 5 and approximately 15% of ethanol, the transmission increases again which is due to ethanol adsorption in ZIF-8. In a similar experiment performed on a glass slide coated only with ZIF-8, a substantial change in transmission begins at 5% ethanol and also 15% (see Fig. 9b, in black), showing that the subsequent increase and decrease in transmission of the stack is caused by the subsequent ethanol loading of the pores of ZIF-90 and ZIF-8. Both curves, for ZIF-8 and for ZIF-8/ZIF-90, flatten out in a plateau above 15% ethanol. This result is in good agreement with the vapor sorption measurements that were performed on powder samples: Ethanol adsorption in ZIF-8 occurs at lower relative pressure compared to the more polar methanol. Therefore, the transmission of the ZIF-8-coated and the ZIF-8/ZIF-90-coated glass is running into a plateau at significantly lower ethanol contents compared to the methanol study shown in the main text, Fig. 6.

7. Cyclic reversibility and stability tests for ZIF-8 and ZIF-90 stacks

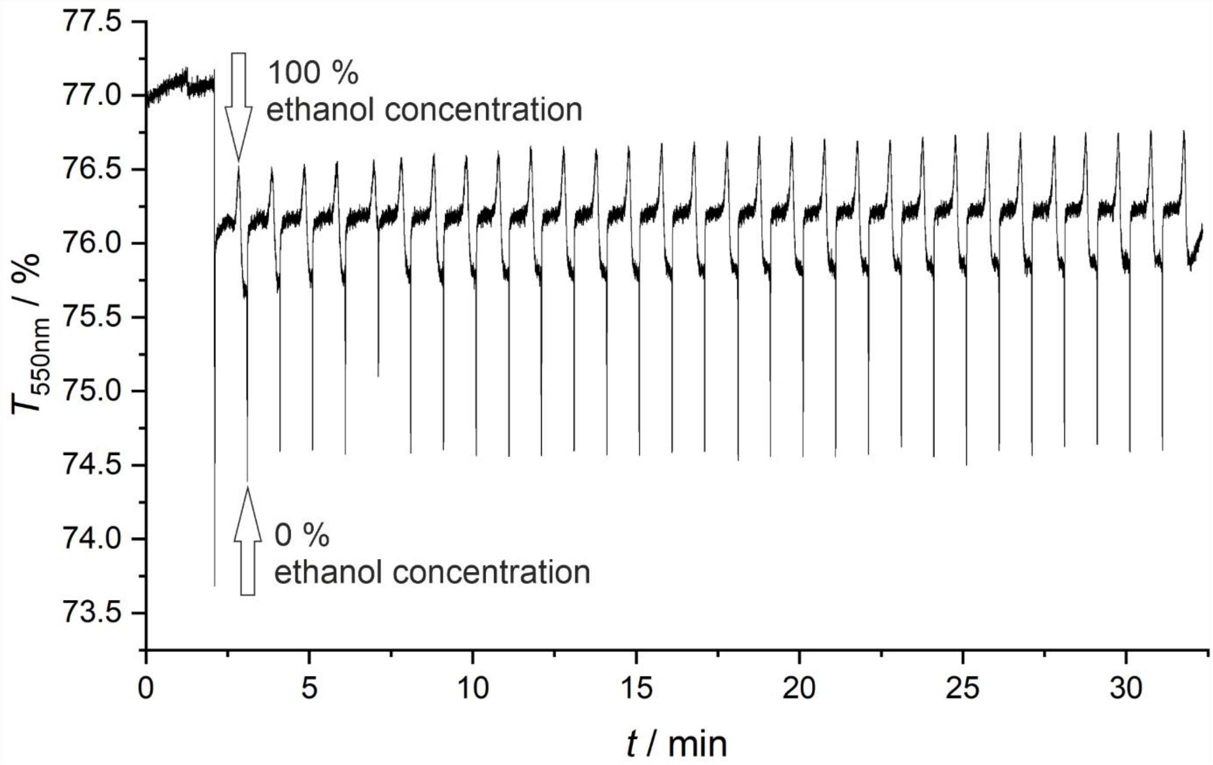

Fig. S17: UV-Vis measurement through a glass slide coated with a ZIF-8/ZIF-90 stack (one seeding layer of ZIF-8 two bilayers of ZIF-90 and ZIF-8) in a gas atmosphere with varying ethanol concentration. Inset points for 100% and 0% relative ethanol concentration are highlighted.

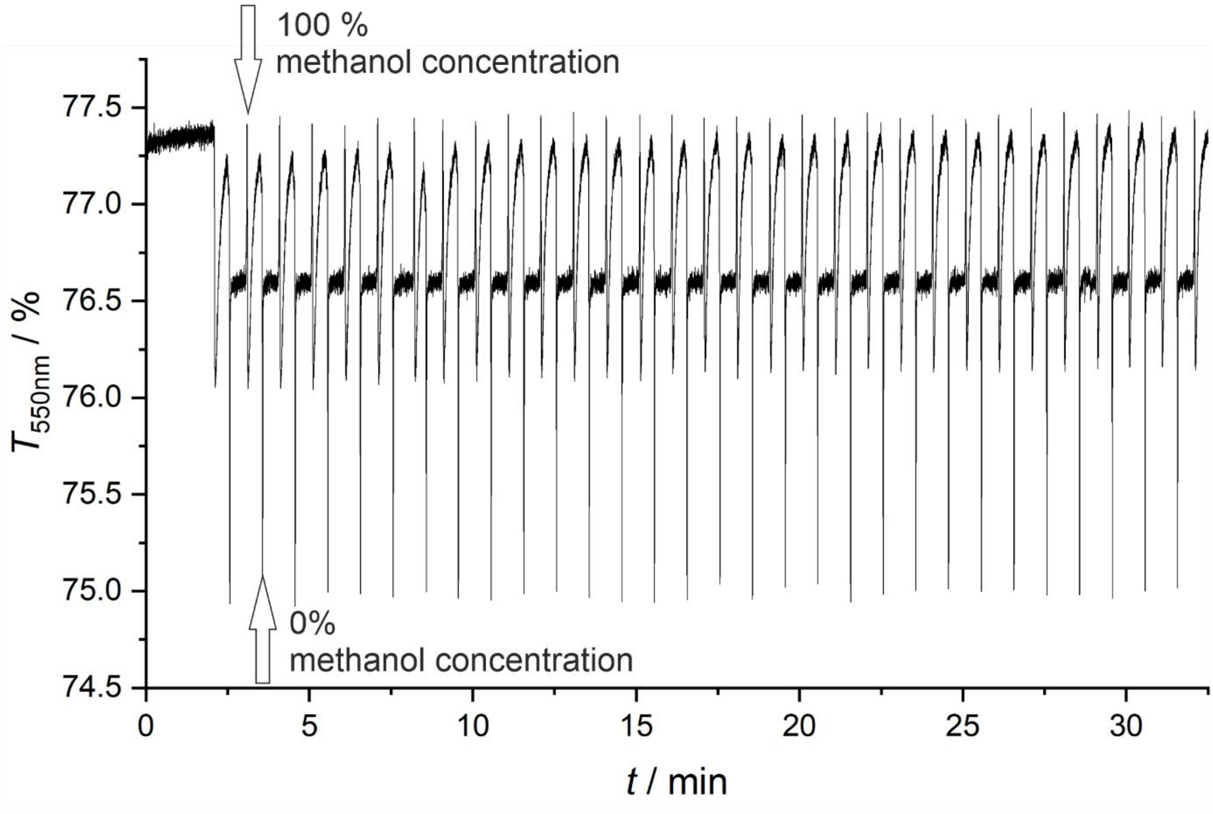

Fig. S18: UV-Vis measurement through a glass slide coated with a ZIF-8/ZIF-90 stack (one seeding layer of ZIF-8 two bilayers of ZIF-90 and ZIF-8) in a gas atmosphere with varying methanol concentration. Inset points for 100% and 0% relative methanol concentration are highlighted.

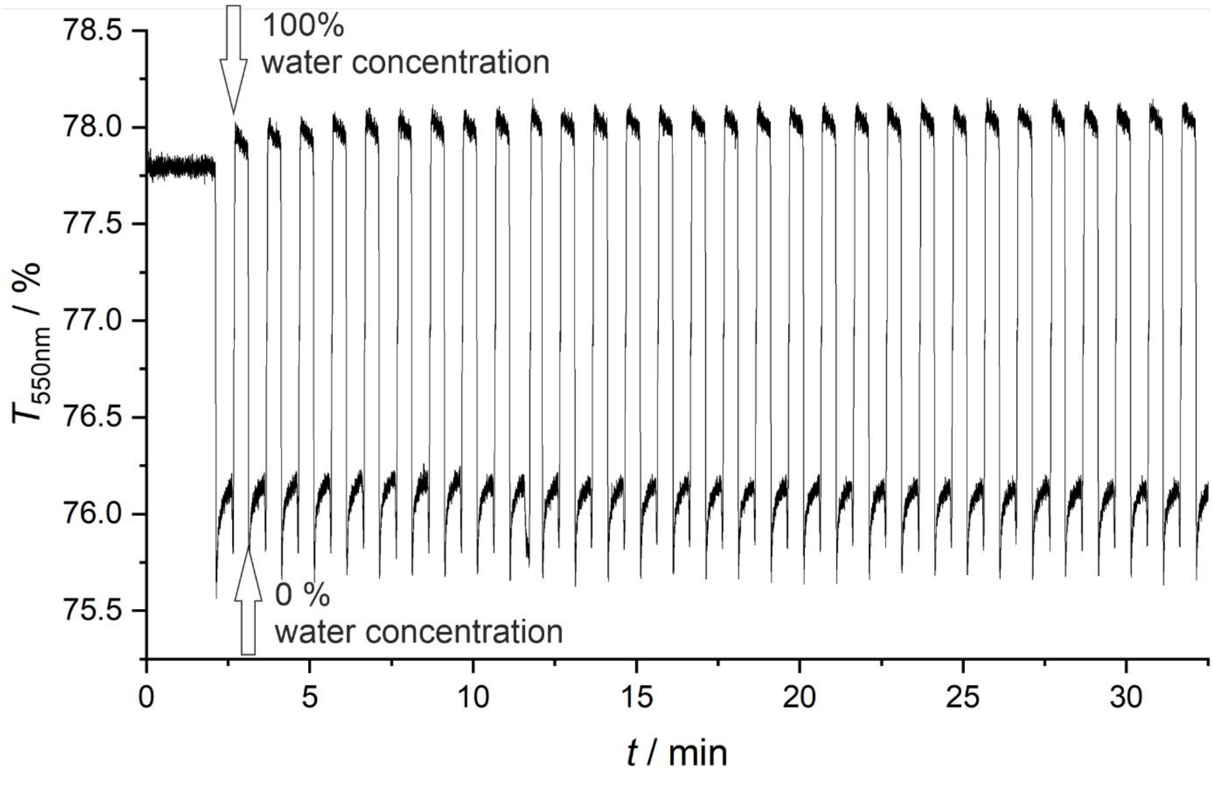

Fig. S19: UV-Vis measurement through a glass slide coated with a ZIF-8/ZIF-90 stack (one seeding layer of ZIF-8 two bilayers of ZIF-90 and ZIF-8) in a gas atmosphere with variable water concentration. Inset points for 100% and 0% relative water concentration are highlighted.

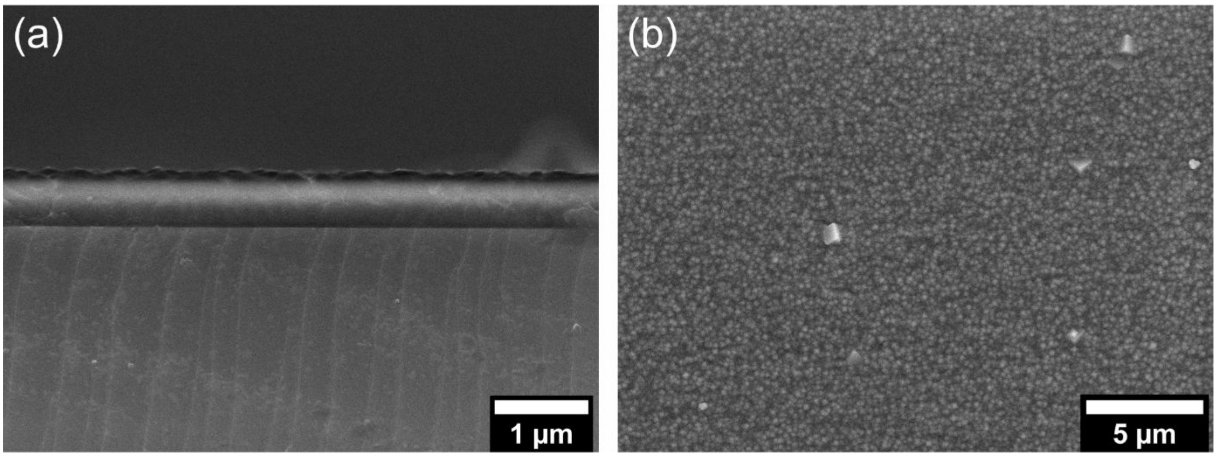

Fig. S20: SEM images of the silicon wafer coated with a ZIF-8/ZIF-90 stack (one seeding layer of ZIF-8 two bilayers of ZIF-90 and ZIF-8) in (a) cross-section and (b) top view. The wafer was used in UV-Vis adsorption measurements with varying concentrations of ethanol, methanol and water. The film remains intact, with no visible cracks or signs of degradation.

8. References

(1) Park, K. S.; Ni, Z.; Côté, A. P.; Choi, J. Y.; Huang, R.; Uribe-Romo, F. J.; Chae, H. K.; O'Keeffe, M.; Yaghi, O. M. Exceptional chemical and thermal stability of zeolitic imidazolate frameworks. *Proc. Natl. Acad. Sci. USA* **2006** *103*, 10186–10191.

(2) Morris, W.; Doonan, C. J.; Furukawa, H.; Banerjee, R.; Yaghi, O. M. Crystals as molecules: postsynthesis covalent functionalization of zeolitic imidazolate frameworks. *J. Am. Chem. Soc.* **2008** *130*, 12626–12627.
